# Differential Root Exudation and Architecture for Improved Growth of Wheat Mediated by Phosphate Solubilizing Bacteria

**DOI:** 10.3389/fmicb.2021.744094

**Published:** 2021-10-15

**Authors:** Mahreen Yahya, Ejaz ul Islam, Maria Rasul, Iqra Farooq, Naima Mahreen, Abdul Tawab, Muhammad Irfan, Lubna Rajput, Imran Amin, Sumera Yasmin

**Affiliations:** ^1^Soil and Environmental Biotechnology Division, National Institute for Biotechnology and Genetic Engineering College, Pakistan Institute of Engineering and Applied Sciences (NIBGE-C, PIEAS), Faisalabad, Pakistan; ^2^Department of Bioindustry and Bioresource Engineering, Sejong University, Seoul, South Korea; ^3^Health Biotechnology Division, National Institute for Biotechnology and Genetic Engineering College, Pakistan Institute of Engineering and Applied Sciences (NIBGE-C, PIEAS), Faisalabad, Pakistan; ^4^Sustainable Agriculture and Food Programme (SAFP), World Wildlife Fund, Khanewal, Pakistan; ^5^Plant Physiology and Biotechnology Agricultural Research Centre, Tandojam, Pakistan; ^6^Agricultural Biotechnology Division, National Institute for Biotechnology and Genetic Engineering College, Pakistan Institute of Engineering and Applied Sciences (NIBGE-C, PIEAS), Faisalabad, Pakistan

**Keywords:** root exudates, organic acids, sugars, amino acids, SOD, POD, CAT, root architecture

## Abstract

Phosphorous (P) deficiency is a major challenge faced by global agriculture. Phosphate-solubilizing bacteria (PSB) provide a sustainable approach to supply available phosphates to plants with improved crop productivity through synergistic interaction with plant roots. The present study demonstrates an insight into this synergistic P-solubilizing mechanism of PSB isolated from rhizosphere soils of major wheat-growing agro-ecological zones of Pakistan. Seven isolates were the efficient P solubilizers based on *in vitro* P-solubilizing activity (233-365 μg ml^–1^) with a concomitant decrease in pH (up to 3.5) by the production of organic acids, predominantly acetic acid (∼182 μg ml^–1^) and gluconic acid (∼117 μg ml^–1^). Amplification and phylogenetic analysis of *gcd*, *pqqE*, and *phy* genes of *Enterobacter* sp. ZW32, *Ochrobactrum* sp. SSR, and *Pantoea* sp. S1 showed the potential of these PSB to release orthophosphate from recalcitrant forms of phosphorus. Principal component analysis indicates the inoculation response of PSB consortia on the differential composition of root exudation (amino acids, sugars, and organic acids) with subsequently modified root architecture of three wheat varieties grown hydroponically. Rhizoscanning showed a significant increase in root parameters, i.e., root tips, diameter, and surface area of PSB-inoculated plants as compared to uninoculated controls. Efficiency of PSB consortia was validated by significant increase in plant P and oxidative stress management under P-deficient conditions. Reactive oxygen species (ROS)-induced oxidative damages mainly indicated by elevated levels of malondialdehyde (MDA) and H_2_O_2_ contents were significantly reduced in inoculated plants by the production of antioxidant enzymes, i.e., superoxide dismutase, catalase, and peroxidase. Furthermore, the inoculation response of these PSB on respective wheat varieties grown in native soils under greenhouse conditions was positively correlated with improved plant growth and soil P contents. Additionally, grain yield (8%) and seed P (14%) were significantly increased in inoculated wheat plants with 20% reduced application of diammonium phosphate (DAP) fertilizer under net house conditions. Thus, PSB capable of such synergistic strategies can confer P biofortification in wheat by modulating root morphophysiology and root exudation and can alleviate oxidative stress under P deficit conditions.

## Introduction

Phosphorus (P) is the second most essential macronutrient after nitrogen (N) for plant growth and development (Shafi et al., 2020). P-deficiency is one of the yield-limiting factors faced by over 40% of agricultural soils worldwide (Elhaissoufi et al., 2020; Brownlie et al., 2021), although P is quite abundant in the soil but mostly present in insoluble forms, rendering a very low concentration (0.1%) of P for plant uptake (Bindraban et al., 2020).

Chemical P fertilizers are produced at a cost of 4 billion USD per year to fulfill the global demand (Adnan et al., 2020; Tewari et al., 2020). However, P use efficacy of applied chemical P fertilizers is as low as 10% throughout the world due to rapid P immobilization by fixation, adsorption, or precipitation reactions with Al^3+^ and Fe^3+^ in acidic soils and with Ca^2+^ in alkaline to normal soils (Penn and Camberato, 2019; Mitter et al., 2021). The excessive and irrational application of chemical fertilizers is not only expensive but also leads to environmental pollution and adverse effects on human health (Manisalidis et al., 2020). Therefore, there is an urgent need to exploit such strategies that can increase P availability for plant uptake from the agricultural soils (Alewell et al., 2020).

Rhizosphere-dwelling phosphate-solubilizing bacteria (PSB) convert insoluble phosphates into bioavailable forms facilitating P uptake by plants via secretion of organic acids (Chungopast et al., 2021). The P solubilizing efficacy of PSB depends on the nature and the amount of organic acid production by these bacteria (Liang et al., 2020). Among different acids produced by PSB, gluconic acid is produced predominately via a direct oxidation pathway of glucose catalyzed by periplasmic glucose dehydrogenase (GDH). This GDH enzyme is encoded by the *gcd* gene and a redox coenzyme pyrroquinolinequinone (PQQ) encoded by *pqq* operon comprising six core genes, *pqq* A, B, C, D, F, and G (An and Moe, 2016). Another enzyme produced by PSB involved in organic P mineralization is phytase encoded by *phy* genes. This enzyme is responsible for P release in soil from organic materials stored in the form of phytate (Richardson and Simpson, 2011).

Besides making soluble P available for plant uptake, P-solubilizing bacteria are involved in plant growth promotion by the production of beneficial metabolites, such as phytohormones like indole acetic acid (IAA), antibiotics or siderophores, aminocyclopropane-1-carboxylate deaminase (ACC), nitrogen fixation, zinc solubilization, and antimicrobial activity against soil-borne plant pathogens (Olanrewaju et al., 2017; Chouyia et al., 2020; Kumawat et al., 2020; Hakim et al., 2021). Furthermore, plant growth-promoting rhizobacteria (PGPR) may indirectly stimulate plant growth and help the plants to alleviate oxidative stress by enhanced production of antioxidant enzymes [e.g., catalase (CAT), peroxidase (POD), and superoxide dismutase (SOD)] in plant tissues (Batool et al., 2020; Bhat et al., 2020; Ha-Tran et al., 2021). Abiotic factors can cause oxidative stress due to an overproduction of reactive oxygen species (ROS). Oxidative product levels like H_2_O_2_ and malondialdehyde (MDA) are the primary marker of ROS and lipid peroxidation responses (Thirupathi et al., 2020). Antioxidant enzymes are of paramount importance to counteract harmful ROS in plant tissues, maintain homeostasis within the cell, and enhance the tolerance of the plant (Dumanović et al., 2020).

With the growing importance as a promising strategy for sustainable agriculture, the mechanism of P solubilization of these PSB-based biostimulants needs to be deeply explored for optimal utilization of these microorganisms under variable field conditions (Alori et al., 2017; Bargaz et al., 2018; Elhaissoufi et al., 2020). It is still difficult to point out a single mechanism responsible for P solubilization by PSB (Sharma et al., 2013). Plant traits, i.e., extensive rooting system and root exudates, can contribute to a greater extent for P uptake. Root architecture is the spatial configuration of the root system in the soil, essential for plant P acquisition (Wissuwa et al., 2020), but root architecture is highly plastic in its response to low P conditions of the soil (Peris et al., 2020). Soil microbes play a key role to build specific patterns of metabolites around the roots by preferentially stimulating root exudation of primary metabolites, i.e., sugars, amino acids, and organic acids. Therefore, soil-dwelling microbes exert a strong influence on plant nutrient sensing (Canarini et al., 2019). Most of the previous studies discussed the direct effects of PSB on plant growth and crop yield (Singh et al., 2018; Liu et al., 2019). Other studies reported beneficial effects of PSB on root parameters (Suleman et al., 2018; Rasul et al., 2019; Rezakhani et al., 2019) showing no stronger linage of root exudation, root architectural variation, and antioxidant enzymes to supplement the plant in response to PSB inoculation. Unfortunately, the relative contribution of PSB to P acquisition remains largely unknown in this symbiotic relationship. Hence, it is imperative to better understand the mechanism of plant–microbe interaction in plant P acquisition under P-limited conditions to reduce reliance on chemical fertilizers.

Therefore, the current study was focused to evaluate the role of native PSB from the wheat rhizosphere of unexplored agro-ecological zones in the establishment of a mutualistic relationship with plants for P acquisition. The experiments were conducted in hydroponics (without the interference of soil) as well as in soil under P-deficient conditions. We hypothesized that (H1) P-solubilizing bacteria enhance nutrient (P) availability, with an increase in root exudations in response to mutualistic association with a plant. We assumed (H2) that PSB inoculation may enhance the activity of antioxidant enzymes and improve root architecture that might help in the adaptation of plants under P-deficiency, and (H3) such PSB-mediated morphological and physiological changes in the plant may improve the grain yield of wheat.

## Materials and Methods

### Sample Collection and Soil Physiochemical Analysis

Rhizosphere soil samples were collected from 31 different sites of eight major wheat-growing agro-ecological zones of Pakistan (25° N∼35° N; 68° E∼74° E) (Supplementary Figure S1). At each sampling site, soils were collected randomly from five different locations at the depth of 0–20 cm, homogenized and stored at 4°C prior to use. A sub-sample of soil (0.5 kg) was used for the determination of physicochemical characteristics (Supplementary Table S1). Soil pH was determined using pH meter (PHS-3C, REX, Shanghai, China; Thomas, 1996). The electric conductivity (EC) of soil was measured using a conductivity meter (DDS-307A, REX; Rhoades, 1993). Soil organic matter was determined using the wet oxidation method (Nelson and Sommers, 1996). Total N was measured using the Kjeldal method (Bremner and Mulvaney, 1982). Soil available P was determined by the sodium bicarbonate (NaHCO_3_) method (Olsen et al., 1954). Exchangeable K and Na were extracted in 1 N ammonium acetate (NH_4_CH_3_CO_2_) solution and estimated by a flame photometer (Model 410, Corning, Halstead, United Kingdom; Simard, 1993).

### Isolation and Identification of P-Solubilizing Bacteria

Phosphate solubilizing bacteria were isolated on NBRIP (National Botanical Research Institute’s Phosphate) (Nautiyal, 1999) and Pikovskaya’s (Pikovskaya, 1948) agar media containing TCP (tricalcium phosphate) as the sole P source. The plates inoculated with soil suspension were incubated at 28°C ± 2°C for 7 days. The colonies with clear halo zone were considered as phosphate-solubilizing isolates and further purified by streaking on NBRIP and Pikovskaya’s agar plates to obtain single colonies. Solubilization index was determined using the formula described by Paul and Sinha (2017).

A biosafety assessment of P-solubilizing isolates was carried out on commercially available blood agar medium. Single colony of bacteria was streaked on blood agar plates and kept at 37°C ± 2°C for 24 h (Russell et al., 2006). Cell and colony morphology, cell motility, and Gram’s staining of PSB were studied using light microscopy (Gopala Krishnamurthy et al., 1967).

Total genomic DNA of each bacterium was obtained using the CTAB method (Wilson, 2001) and was used as a template for amplification of 16S rRNA gene using universal primers (forward PA 5′-AGACTTTGATCCTGCTCAG-3′ and reverse PH 5′-AGGAGGTGATCCAGCCGCA-3′) according to Ayyaz et al. (2016). Amplified 16S rRNA PCR products were analyzed on 1% agarose gel and further purified by PCR purification kit (QIAGEN Sciences, Germantown, MD, United States) prior to sequencing (Macrogen Inc., Seoul, South Korea). The derived sequences were compared to 16S rRNA gene sequences available at NCBI Gen Bank database using the BLAST algorithm for bacterial identification. The nucleotide sequences were aligned, and a phylogenetic tree was constructed using the maximum likelihood method (Kumar et al., 2016). Sequences were deposited to NCBI GenBank^1^.

### Quantitative P Solubilization and Production of Organic Acids

Quantification of soluble P and organic acids released by PSB was carried out in an *in vitro* assay at 7 DPI (days post-inoculation). A single purified colony of each isolate was inoculated in NBRIP and Pikovskaya’s broth media. Inoculated broth cultures were incubated at 28°C ± 2°C and 180 rpm on the shaker. Cultures were subjected to centrifugation at 4,000 rpm for 10 min at 4°C to get cell-free supernatants. The available P in culture-supernatant was spectrophotometrically estimated at 880 nm using the molybdenum blue method (Murphy and Riley, 1962).

For analysis of organic acids, NBRIP filtrates were quantified on high-performance liquid chromatography (HPLC; Agilent 1,200 Series; Agilent Technologies, Santa Clara, CA, United States) equipped with C-18 column using mobile phase methanol: phosphate buffer (90: 10 v/v; pH 2.7) at flow rate of 1 ml min^–1^ and monitored at 210 nm (Tahir et al., 2013). Organic acids, i.e., acetic, citric, gluconic, malic, and succinic acid, were analyzed. Peak area and retention time were compared to standards (Sigma) for the quantification of organic acids (Vyas and Gulati, 2009).

### Amplification of Genes Responsible for P Solubilization

The glucose dehydrogenase (*gcd*) gene of PSB was amplified by reported primers, forward 5′-GACCTGTGGGACATGGACGT-3′ and reverse 5′-GTCCTTGCCGGTGTAGSTCATC-3′ (Chen et al., 2016). Amplification of *pqqE* gene was carried out by degenerate primers, forward 5′-TTYTAYACCAACCTGATCACSTC-3′ and reverse 5′-TBAGCATRAASGCCTGRCG-3′ (Perez et al., 2007). Phytase (*phy*) gene was amplified by reported primers, forward 5′-ACAGACACGAAGTGACCTACC-3′ and reverse 5′-CCAAGCAGACGAGAATCC-3′ (Han et al., 2008). PCR product was purified and sequenced by Macrogen (Rockville, MD, USA). Sequence data were aligned and compared to published sequences at NCBI (see text Footnote 1) using BLASTX^2^. Phylogenetic analysis was performed by maximum likelihood method using MEGA6 software (Kumar et al., 2016).

### Plant Growth-Promoting Traits of PSB

Plant growth-promoting traits of PSB were evaluated by using standard protocols. Indole acetic acid (IAA) production by PSB was quantified by using Salkowski’s method (Gordon and Weber, 1951) on HPLC (Tien et al., 1979). Gibberellic acid produced by PSB was quantified according to protocol by Tien et al. (1979).

Siderophore production by PSB was detected using chrome auzurol S (CAS) agar medium (Schwyn and Neilands, 1987). The development of pink coloration around the bacterial colonies is an indication of siderophore production. Zinc solubilization by PSB was determined on Tris–minimal salts medium supplemented with insoluble zinc oxide (ZnO: 14 mM). Formation of the halo zone around bacterial colonies showed zinc solubilization. Solubilization index was measured as described by Fasim et al. (2002).

### Effect of PSB Inoculation on Wheat Germination and Seedling Vigor

Based on *in vitro* P solubilizing efficacy, seven P-solubilizing isolates (ZW9, ZW32, SSR, D1, S1, TJA, and TAYB) were selected to study their effect on seed vigor index, germination, and root morphological parameters. Seeds of wheat variety “Faisalabad-08” were surface-sterilized for 5 min with 1.5% sodium hypochlorite solution followed by successive washings with sterile water to remove disinfectant traces. Sterilized seeds were inoculated with bacterial cultures (1 × 10^9^ CFU ml^–1^) separately for 30 min. Uninoculated seeds soaked in LB broth were used as controls. Seeds were kept on water agar (0.25%) containing petri dishes (9 cm diameter × 2 cm depth). The plates were incubated in dark for 72 h at 28°C ± 2°C followed by 12-h dark and light cycles for 7 days in a growth room. Six biological replicates and 15 seeds per plate for each treatment were arranged in completely randomized design (CRD). The experiment was conducted twice, and different plant growth parameters were studied at 7 days post inoculation (DPI).

Percent germination and vigor index of freshly harvested seedling was measured (Islam et al., 2016). Root growth parameters including root length (cm), root volume (cm^3^), area of projection (cm^2^), length per volume (cm m^–3^), root diameter (cm^2^), root surface area (cm^2^), tips, forks, and crossings were studied using rhizoscanner (Epson, Los Alamitos, CA, United States) equipped with software WinRHIZO (Regent Int. Dev., Ltd., Richmond, BC).

### Development of PSB Consortia

Seven efficient PSB (ZW9, ZW32, SSR, D1, S1, TJA, and TAYB) selected for consortia development were studied for *in vitro* compatibility test (Irabor and Mmbaga, 2017). Bacterial inoculum was spotted on nutrient agar plate spread with the tested strain. The plates were kept at 28°C ± 2°C and observed up to 72 h. No inhibition zone was observed around any bacterial colony, indicating that they were compatible with each other.

Three different consortia were prepared using two efficient PSB from their respective native soil while the most efficient phosphobacteria, i.e., SSR was added in all three consortia. Consortium-1, comprising *Enterobacter* spp. ZW9, ZW32, and *Ochrobactrum* sp. SSR, was developed for wheat variety 1 (Faisalabad-08) recommended for Province 1 (Punjab). Consortium-2 (*Pantoea* sp. S1, *Enterobacter* sp. D1, and *Ochrobactrum* sp. SSR) was used for wheat variety 2 (Fakhr-e-Sarhad) recommended for Province 2 (Khyber Pakhtunkhwa). Consortium-3 (*Ochrobactrum* sp. SSR, *Pseudomonas* sp. TJA, and *Bacillus* sp. TAYB) was used for wheat variety 3 (Benazir-13) recommended for Province 3 (Sindh).

A single colony of each bacterium grown separately in LB medium at 28°C ± 2°C for 24–48 h. An equal volume of each bacterium grown in LB was mixed to develop a consortium (1 × 10^9^ CFU ml^–1^). Seeds were surface-sterilized with sodium hypochlorite solution (1.5%) for 5 min followed by successive washings (six times) with sterile water to remove disinfectant traces. Sterilized seeds of wheat variety 1, 2, and 3 were soaked in consortium-1, consortium-2, and consortium-3 suspensions (1 × 10^9^ CFU ml^–1^); respectively for 30 min. Uninoculated seeds soaked in sterilized LB broth were used as controls.

### Hydroponic Experiment to Study the Effect of PSB on Root Exudation and Anti-oxidant Enzymes

**FIGURE 1:**
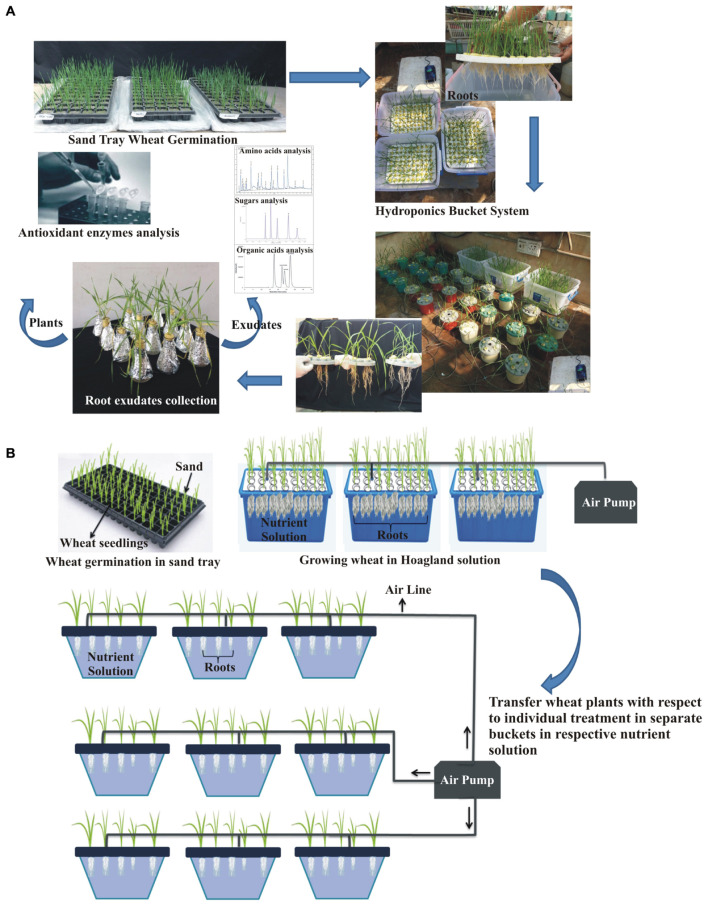
Illustration of the standing-aerated hydroponic growing system consisting of experimental setup for root exudates collection and analysis **(A)**. Demonstration of sterilized hydroponic system; each treatment was aerated individually **(B)**.

A hydroponic experiment was conducted to study the inoculation effects on wheat root exudation and anti-oxidant production in P-deficient conditions under greenhouse at NIBGE, Faisalabad (31°25′0″N 73°5′28″E). Surface-sterilized seeds were inoculated as described in the previous section. Inoculated seeds of variety 1, 2, and 3 and their respective uninoculated seeds were germinated in moistened sand with relative humidity 60–70% and day/night temperature of 25°C/23°C in the green house (Figure 1). After 1 week, seedlings were transferred to experiment buckets (24 cm × 37 cm) each having a volume of 4.5 L filled with half-strength Hoagland solution (Hoagland and Arnon, 1950) for proper plant growth and development (Islam et al., 2008).

Subsequently, 2-week-old plants were transferred to an aerated hydroponic system comprised of the opaque plastic box (13 cm × 13 cm) each having a volume of 1.5 L, filled with treatment-wise Hoagland solutions (Hoagland and Arnon, 1950). Three biological treatments, i.e., T1: Consortia-inoculated plants supplemented with modified hoagland containing 1 g L^–1^ tricalcium phosphate (TCP) as P-deficient solution, T2: Positive control (uninoculated plants supplemented with Hoagland containing all nutrients including P), and T3: Negative control (uninoculated plants supplemented with modified Hoagland containing 1 g L^–1^ TCP as P-deficient solution) were arranged in a two-factorial completely randomized design (CRD). Each box was aerated with an adjustable air pump that operates continuously to ensure a sufficient supply of air. Five seedlings per box were grown under a daily temperature regime of 25°C ± 2°C per day and 18°C ± 2°C per night with a photoperiod of 12-h light and darkness. Hoagland solution from each treatment was changed after every seventh day (Somasegaran and Hoben, 2012).

The plants were harvested 45 days after cultivation in a hydroponic system, and data were collected to study growth parameters of the plants, i.e., fresh and dry weight, root-shoot length, and plant P. Root growth parameters including root length (cm), root volume (cm^3^), area of projection (cm^2^), length per volume (cm m^–3^), root surface area (cm^2^), root diameter (cm^2^), tips, forks, and crossings were studied using rhizoscanner (Epson photo scanner V700) equipped with software WinRHIZO (Regent Int. Co., Ltd.).

### Collection and Analysis of Root Exudates

Wheat plants were harvested after 45 days of cultivation in hydroponics, and root exudates were extracted in an Erlenmeyer glass flask by immersing the plant roots in sterile 2 mM CaCl_2_ solution (Vranova et al., 2013). Flasks were wrapped with aluminum foil and placed in sunlight for 4 h. Root exudates collected in CaCl_2_ solution were filtered using a 0.2-μm spin-filter (Thermo Fisher Scientific, Waltham, MA, United States) and stored at −20°C for composition analysis (Yang et al., 2006).

#### Determination of Amino Acids

Aliquots (10 ml) of root exudates were lyophilized, cleaned with cold acetone, and kept overnight at −20°C to remove protein interference. The supernatant was transferred to new tubes, vacuum-dried, and filtered through 0.2-μm spin-filter (Thermo Fisher Scientific). To derivatize amino acids, 5 μl OPA-3-MPA (O-pthaladehyde 3-mercaptopropionic acid) and 7 ml borate buffer (0.4 M) were added to a 1-ml sample and kept at room temperature for 1 min prior to run. Amino acids were separated in reversed-phase HPLC (Agilent 1200 Series) supplemented with a C-18 guard column at 1 ml min^–1^ flow rate and detected (λexc 250 nm; λem 395 nm) with a fluorescence detector (Frank and Powers, 2007).

#### Determination of Sugars

Aliquots (10 ml) of root exudates were lyophilized, re-dissolved in 40 μl H_2_O, and filtered through a 0.2-μm spin-filter (Thermo Fisher Scientific). Sugars were separated by HPLC under isocratic conditions (acetonitrile and water in 75: 25 v/v) at 1 ml min^–1^ flow rate (Lugtenberg et al., 1999) and detected at 487 nm using a UV detector.

#### Determination of Organic Acids

Organic acids in root exudates were quantified by following the procedure as described by Kamilova et al. (2006). Organic acids were separated using mobile phase 10 mM H_3_PO_4_ at 0.8 ml min^–1^ flow rate and detected at 210 nm using HPLC with a UV detector (Agilent 1200 Series).

#### Determination of Antioxidant Enzyme Activity

Fresh leaves (1 g) of wheat plants harvested from the hydroponic system were homogenized in ice-cold 10 ml of potassium phosphate buffer (100 Mm; pH 7) for extraction of enzymatic anti-oxidants. The homogenate was centrifuged (10,000 × g) for 20 min at 4°C and stored at −80°C for analysis of antioxidant enzymes.

#### Superoxide Dismutase (SOD)

SOD activity was determined by following the process described by Dhindsa and Matowe (1981). The reaction mixture contained 50 μl Nitro blue tetrazolium chloride (NBT), 100 μl L-methionine, 50 μl riboflavin, 250 μl sodium phosphate buffer, and 50 μl enzyme extract. The reaction mixture was placed under a fluorescent lamp (3 W) for 15 min followed by exposure to dark for 15 min and measured the absorbance at 560 nm using spectrophotometer (CamSpec M550 double beam UV-Vis; Spectronic Camspek Ltd, Leeds, United Kingdom). One unit of SOD activity is the amount of enzyme required for 50% inhibition of photo-chemical reduction of NBT.

#### Catalase (CAT)

CAT activity was measured as described by Aebi (1984). The reaction mixture contained 1.9 ml phosphate buffer (50 mM; pH 7) and the enzyme-extract (100 μl). To initiate the reaction, 1 ml H_2_O_2_ was added to the reaction mixture the absorbance was measured at 240 nm after every 30 s until 3 min using spectrophotometer. One unit of CAT activity is defined as absorbance change of 0.01 U min^–1^.

#### Peroxidase (POD)

POD activity was measured by mixing 50 mM phosphate buffer (pH 7), 20 mM of guaiacol, and 100 μl of enzyme extract (Rao et al., 1996). To initiate the reaction, 40 mM H_2_O_2_ was added to the reaction mixture and incubated at 25°C for 5 min. Change in the absorbance at 436 nm was monitored every 2 min. One unit of POD activity is an absorbance change of 0.01 U min^–1^. All antioxidants are expressed as unit mg^–1^ fresh protein.

#### Malondialdehyde (MDA) and H_2_O_2_

Change in lipid peroxidation was studied by calculating MDA production in leaves of wheat plants harvested from the hydroponic system to determine the level of oxidative damage caused by P deficiency. Fresh leaves (1 g) were homogenized in 20 ml trichloroacetic acid (TCA; 0.1 percent) and centrifuged (12,000 × *g*) for 20 min at 4°C for MDA and H_2_O_2_ contents estimation.

For MDA extraction aliquot of supernatant (1 ml) was mixed with 4 ml TCA containing thiobarbituric acid (TBA; 5%). The reaction mixture was incubated at 95°C for 15 min and subsequently cooled on ice-bath to stop the reaction. Samples were centrifuged at 12000 × *g* for 10 min, and absorbance was recorded at 532 nm and 660 nm (Demiral and Türkan, 2005).

H_2_O_2_ content was measured according to Velikova et al. (2000). The reaction mixture (3 ml) contains 0.5 ml supernatant, 10 mM potassium phosphate buffer (pH 7), and 1 M KI (potassium iodide) solution. The mixture was vortexed, and absorbance was monitored at 390 nm. Amount of H_2_O_2_ content was calculated using a standard curve prepared with known H_2_O_2_ concentrations.

### Pot Experiment Using Soils of Different Agro-Ecological Zones

Effects of P-solubilizing consortium-1, consortium-2, and consortium-3 on recommended wheat varieties of Province 1, 2, and 3 were further assessed in a pot experiment at NIBGE (31°25′0″N 73°5′28″E) greenhouse using native soils collected from eight different agro-ecological zones of Pakistan. Among 31 sites of isolation (Supplementary Figure S1 and Supplementary Table S1), soils from 16 major wheat-growing sites were selected for evaluation of PSB consortia (Supplementary Table S5).

Consortium-1 was used for wheat variety 1 (Faisalabad-08) recommended for sites (site no. 1–9, Supplementary Table S5) of Province 1. Consortia-2 was used for wheat variety 2 (Fakhr-e-Sarhad) recommended for sites (site no. 1–3, Supplementary Table S5) of Province 2. Consortia-3 was applied on wheat variety 3 (Benazir-13) recommended for sites (site no. 1–4, Supplementary Table S5) of Province 3 (Supplementary Table S5). Seeds were surface-sterilized with sodium hypochlorite solution (1.5%) as described in previous sections. Sterilized seeds were inoculated with respective PSB consortia (1 × 10^9^ CFU ml^–1^) for 30 min. Uninoculated seeds dipped in LB medium were used as controls. Sowing was done in pots (10 cm diameter) containing 300 g soil per pot (supplemented with TCP @ 1 g/pot) from 16 different wheat-growing areas of three provinces. There were 32 treatments (16 different soils with and without inoculation) and six biological replicates arranged in a two-factor completely randomized design (CRD; Supplementary Table S5). For proper seed germination, all pots were watered before sowing. Each pot has two plants. After seed germination, pots were kept moist as per requirement by providing them with water and nutrient solution (Hoagland and Arnon, 1950) without P source, alternatively.

Plants were uprooted at 45 DAS and evaluated for the growth parameters, i.e., plant fresh weight, dry weight, root length, and shoot length. Ground plant material (1 g) from each treatment was used for plant P analysis by using the tri-acid digestion method as described by Tandon (1993). Rhizospheric soils from each treatment were analyzed for viable count (Somasegaran and Hoben, 2012). Soil available P was measured by the sodium bicarbonate (NaHCO_3_) method (Olsen et al., 1954), and soil phosphatase activity was measured by the p-nitophenyl method as described by Tabatabai and Bremner (1969).

### Evaluation of PSB Consortium for Wheat Yield Parameters

Based on the higher P-solubilizing potential of consortium-1 under controlled conditions, a pot experiment was carried out to evaluate the effects of consortium-1 on yield and P content of wheat under net house conditions at NIBGE, Faisalabad (31°23′45.1″N, 73°01′3.4″E) during the wheat season November 2018–April 2019.

Seeds were pelleted by mixing press mud as carrier material (@ 50 kg seed per kg carrier material) with consortium-1 (1 × 10^9^ CFU ml^–1^) and left for 1 h. Uninoculated pelleted seeds were used as a control. Press mud-based carrier material had N 4.3%, P 2%, organic-matter 65%, pH 7.5, EC 3 ms cm^–1^, 40% moisture content, and 2 mm particle size. Sowing was done in earthen pots (diameter: 30 cm) filled with 12 kg soil (Loam texture, organic-matter 0.57%, pH 8.2, and available P: 1.9 mg kg^–1^) and watered for proper seed germination before sowing. There were three treatments including consortium-1 inoculated seeds supplemented with 80% recommended dose of DAP (i.e., 20% reduced DAP) and two uninoculated controls supplemented with 80 and 100% DAP (i.e., recommended dose of DAP), respectively. Six biological replicates and six plants per pot were arranged in two-factor CRD.

Recommended doses of fertilizers were applied (N: P, 15:100 kg ha^–1^). P was added in the form of DAP at the time of sowing, while N (urea) was added in split doses, i.e., initially at the time of sowing and subsequently with first and second irrigations. Plants were uprooted at 35 DAS and the growth parameters, i.e., root length, shoot length, and plant dry weight were recorded. At maturity, plants were harvested and data for grain yield, plant biomass, plant height, number of tillers, and plant P were recorded (Tandon, 1993). Rhizosphere soil from each treatment was analyzed for available P (Olsen et al., 1954) and phosphatase activity (Tabatabai and Bremner, 1969).

### Detection of Inoculated PSB

The rhizosphere soil of wheat plants grown in pots was also evaluated for the survival of inoculated PSB using viable count (Somasegaran and Hoben, 2012) and BOX-PCR (Basheer et al., 2016). Re-isolated PSB was identified by comparing the morphological characteristic of inoculated bacteria and other plant growth-promoting attributes like P solubilization, IAA production, and zinc solubilization (Yasmin et al., 2016). Strain-specific fingerprints of re-isolated PSB were compared to those of pure colonies using BOX-A1R primer 5′ CTACGGCAAGGCGACGCTGACG 3′ (Basheer et al., 2016).

### Statistical Analysis

All data from *in vitro* studies, HPLC results, and pot experiments were analyzed statistically by ANOVA. The variation between treatments was compared by least significant difference (LSD) at 1 and 5% level of confidence for lab and net house experiments using Statistix 10 software (Analytical Software, Tallahassee, FL, United States). Principal component analysis (PCA) for various root and yield parameters and regression analysis were carried out using SPSS 23.0 software (SPSS Inc., Chicago, IL, United States). Box plots were applied for analysis of soil parameters in a pot experiment using different soils according to ANOVA and Tukey’s HSD test at *p* < 0.05) using Origin Software Package Version 2020b (OrginLab Corporation, Northampton, MA, United States).

## Results

### Isolation and Identification of PSB

**FIGURE 2:**
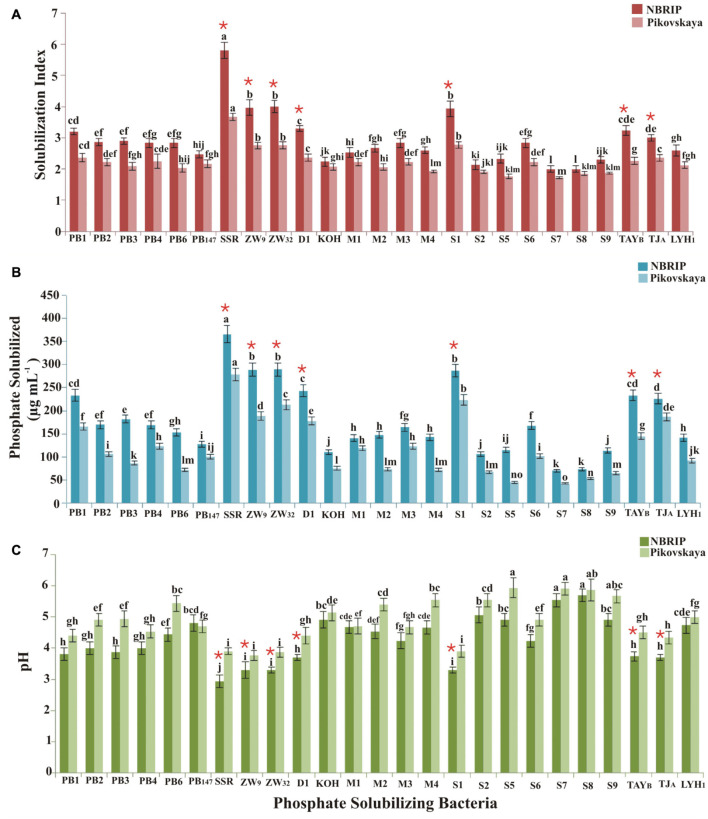
Mineral phosphate solubilization by PSB isolated from different wheat-growing agro-ecological zones of Pakistan. P solubilizing index was calculated from halo zone formation on NBRIP and Pikovskaya’s agar medium **(A)**. Quantification of P solubilized by PSB in liquid NBRIP and Pikovskaya’s medium **(B)**. pH of medium (initial pH 7) at seventh day of pot inoculation **(C)**. There were six biological replicates, and standard error of the means is represented as bars. The significant difference (*p* < 0.01) among treatments is denoted as means and represented by different letters (a, b, c).*PSB showing significantly higher P solubilizing index and P solubilized, while significantly lowering the pH of the NBRIP and Pikovskaya’s medium. ZW9, ZW32, and D1 are *Enterobacter* spp.; SSR: *Ochrobactrum* sp.; S1: *Pantoea* sp.; TJA: *Pseudomonas* sp.; and TAYB: *Bacillus* sp.

Wheat rhizospheric soil samples were collected from eight different agro-ecological zones of Pakistan for isolation of PSB (Supplementary Figure S1). Among 377 bacterial isolates, 25 strains (PB1, PB2, PB3, PB4, PB147, SSR, ZW9, ZW32, D1, KOH, M1, M2, M3, M4, S1, S22, S5, S6, S7, S8, S9, TAYB, TJA, and LYH1) were efficient P-solubilizers, indicated by the formation of halo zone on NBRIP and Pikovskaya’s agar media. The solubilization index (SI), ranging from 2.1 to 5.8 on NBRIP medium and 1.1 to 2.9 on Pikovskaya’s agar medium, showed the P solubilization potential of bacteria (Figure 2A).

Among 25 selected PSB, *Ochrobactrum* sp. SSR (SI: 5.8), *Enterobacter* sp. D1 (SI: 3.8), *Pantoea* sp. S1 (SI: 4), *Pseudomonas* sp. TJA (SI: 3.6), and *Bacillus* sp. TAYB showed a higher P solubilization index on NBRIP medium (Figure 2A). Light microscopy showed that these PSB were motile rod-shaped except few cocci (*Enterobacter* spp., strains PB1, D1 and S8) and were Gram negative (except *Bacillus* spp., S6 and TAYB). 16S rRNA gene identification revealed that these bacteria belonged to genera *Acinetobacter*, *Bacillus, Enterobacter*, *Ochrobactrum*, *Pantoea*, *Pseudomonas*, and *Stenotrophomonas* (Accession numbers MN754080, MN754081, MN754082, MN860090 to MN860102, MK422612 to MK422620, and MK817561 (Supplementary Table S2).

### Quantitative P Solubilization and Production of Organic Acids

All PSB strains showed P solubilization activity in both NBRIP and Pikovskaya’s broth along with a subsequent decrease in pH (Figure 2C). The solubilized P ranged from 69–365 μg ml^–1^ in NBRIP medium with decline in pH up to 3.6, and from 43 to 278 μg ml^–1^ P in Pikovskaya’s medium with pH decline up to 4, respectively. Maximum release of P (365 μg ml^–1^) was observed for *Ochrobactrum* sp. SSR in NBRIP medium (Figure 2B). Primary selection of seven PSB isolates (*Enterobacter* sp. ZW9, *Enterobacter* sp. ZW32, *Ochrobactrum* sp. SSR, *Enterobacter* sp. D1, *Pantoea* sp. S1, *Pseudomonas* sp. TJA, and *Bacillus* sp. TAYB) was carried out based on their P-solubilizing potential as indicated by *in vitro* qualitative and quantitative tests.

Organic acids released in liquid culture during P solubilization by PSB strains were acetic acid (20–182 μg ml^–1^), gluconic acid (20-117 μg ml^–1^), malic acid (20-117 μg ml^–1^), and oxalic acid (1-16 μg ml^–1^). Citric acid (10.5-82 μg ml^–1^) was produced by *Enterobacter* spp. ZW9 and ZW32, *Pseudomonas* sp. TJA, *Bacillus* sp. TAYB, and *Ochrobactrum* sp. SSR. Succinic acid (4-72 μg ml^–1^) was produced by *Enterobacter* spp. ZW9, ZW32, D1, *Pseudomonas* sp. TJA, *Pantoea* sp. S1, and *Ochrobactrum* sp. SSR (Table 1).

**TABLE 1:** Plant growth-promoting traits and organic acids produced by phosphate-solubilizing bacteria.

Phosphate-solubilizing bacteria	^1^Organic acids produced (μg ml^–1^)						Plant growth-promoting traits			
	Acetic acid	Citric acid	Gluconic acid	Malic acid	Oxalic acid	Succinic acid	^2^IAA Production (μg ml^–1^)	^3^Siderophore Production	^4^Zinc Solubilization (SI)	^5^Gibberellic acid (μg ml^–1^)
*Enterobacter* sp. ZW9	156 ± 7.8 C	10.5 ± 0.5 C	112 ± 5.5 C	30 ± 1.5 C	10 ± 0.5 C	68 ± 3.4 B	37.8 ± 1.9	+++	3.2 ± 0.2	5.2 ± 0.3 F
*Enterobacter* sp. ZW32	144 ± 7.2 D	18.0 ± 0.9 B	130 ± 6.0 A	71 ± 3.5 B	8 ± 0.5 D	62 ± 3.0 C	31.7 ± 1.6	+++	3.4 ± 0.2	17.0 ± 0.8 B
*Ochrobactrum* sp. SSR	182 ± 9.0 A	82.0 ± 4.0 A	117 ± 5.5 B	82 ± 4.0 A	16 ± 0.8 A	72 ± 3.0 A	48.0 ± 2.4	++++	4.0 ± 0.2	32.5 ± 1.6 A
*Enterobacter* sp. D1	161 ± 8.0 BC	11.0 ± 0.5 C	25 ± 1.2 E	5.0 ± 0.2 E	5.0 ± 0.3 E	13.5 ± 0.5 D	8.5 ± 0.4	+++	3.0 ± 0.2	13.4 ± 0.6 C
*Pantoea* sp. S1	169 ± 6.5 B	16.0 ± 0.8 B	33 ± 1.6 D	4.9 ± 0.3 E	11.2 ± 0.5 B	7.5 ± 0.3 E	8.7 ± 0.4	++	3.2 ± 0.2	16.5 ± 0.8 B
*Bacillus* sp. TAYB	64 ± 3.0 E	12.5 ± 0.6 C	32 ± 1.6 D	23.5 ± 1.2 D	1.0 ± 0.05 G	4.7 ± 0.2 F	4.8 ± 0.2	+	2.9 ± 0.1	11.5 ± 0.5 D
*Pseudomonas* sp. TJA	20 ± 1.0 F	11.5 ± 0.5 C	20 ± 1.0 F	0.5 ± 0.02 F	3.0 ± 0.1 F	4.0 ± 0.2 F	3.9 ± 0.2	+	2.8 ± 0.1	9.8 ± 0.4 E

*^1^Organic acids produced by phosphate-solubilizing bacteria in NBRIP broth medium at 7^th^ day were analyzed on High Performance Liquid Chromatography (HPLC).*

*^2^Indole acetic acid (IAA) was quantified by HPLC.*

*^3^Siderophore-producing activity of PSB was qualitatively studied by using universal CAS assay. + represents level of siderophore production.*

*^4^Zinc solubilization detected by plate assay; SI, solubilization index.*

*^5^Gibberellic acid was quantified by HPLC. All values are an average of six biological replicates. ± represents standard deviation. Means followed by the same letter differ non-significantly at p = 0.01 according to LSD.*

### Amplification of Genes Responsible for P Solubilization

**FIGURE 3:**
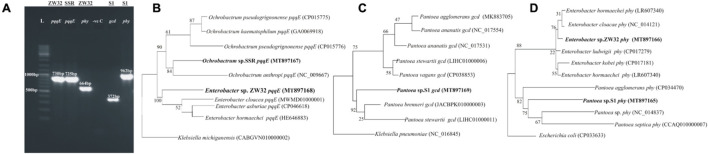
Amplification and sequence-based phylogenetic trees of *pqqE*, glucose dehydrogenase (*gcd*), and phytase (*phy*) genes of *Enterobacter* sp. ZW32, *Ochrobactrum* sp. SSR, and *Pantoea* sp. S1. Gel photograph indicating amplification of *pqqE*, *gcd*, and *phy* genes **(A)**. Sequence-based phylogenetic trees of *pqqE* gene **(B)**, *gcd* gene **(C)**, and *phy* genes **(D)** were constructed by maximum likelihood method.

Glucose dehydrogenase gene (*gcd*) and phytase (*phy*) were amplified in *Pantoea* sp. S1 by conserved PCR primers. Amplification of *pqqE* gene using degenerate primers was observed for *Ochrobactrum* sp. SSR and *Enterobacter* sp. ZW32 (Figure 3). Phylogenetic analysis of partial DNA sequence of *pqqE* gene for *Ochrobactrum* strain SSR (GenBank accession number MT897168) and *Enterobacter* sp. ZW32 (GenBank accession number MT897167) exhibited 92 and 95% identity with *pqqE* gene of *Ochrobactrum pseudogrignonense* strain (CP015776) and *Enterobacter cloaceae* (MWMD01000001), respectively. *Gcd* gene amplified from *Pantoea* strain S1 (GenBank accession MT897169) showed 92% identity to *gcd* gene of *Pantoea brenneri* (CP034148). *phy* genes amplified from *Enterobacter* sp. ZW32 (GenBank accession MT897166) and *Pantoea* strain S1 (GenBank accession MT897165) showed 96% homology to *phy* gene of *Enterobacter ludwigii* (CP017279) and *Pantoea agglomerans* strain (CP034470), respectively (Supplementary Table S3).

### Plant Growth-Promoting Traits of PSB

The most efficient seven P-solubilizing strains produced IAA (2.6–48 μg ml^–1^) in LB medium supplemented with tryptophan. HPLC analysis showed maximum IAA production by *Ochrobactrum* sp. SSR (48 μg ml^–1^) followed by *Enterobacter* sp. ZW32, (31.7 μg ml^–1^), *Enterobacter* sp. ZW9 (13 μg ml^–1^), *Pantoea* sp. S1 (8.5 μg ml^–1^), and *Enterobacter* sp. D1 (8.6 μg ml^–1^). Gibberellic acid (9.8–32 μg ml^–1^) was produced by all tested strains (Table 1).

Among studied P-solubilizing bacteria, i.e., *Ochrobactrum* sp. SSR, *Enterobacter* sp. ZW9, *Pantoea* sp. S, *Enterobacter* sp. ZW32, *Enterobacter* sp. D1, *Pseudomonas* sp. TJA, and *Bacillus* sp. TAYB produced siderophores and solubilized zinc oxide with a solubilization index ranging from 2.8 to 4 (Table 1). PSB showed no halo zone formation on blood agar medium as compared to control and was used for further studies.

### Effect of PSB on Wheat Germination and Seedling Vigor

The plate germination assay showed the positive effect of P-solubilizing bacteria (*Enterobacter* spp. ZW9 and ZW32, *Ochrobactrum* sp. SSR, *Enterobacter* sp. D1, *Pantoea* sp. S1, *Pseudomonas* sp. TJA, and *Bacillus* sp. TAYB) on wheat seedlings. A maximum vigor index (2886 ± 68.17) was found for seedlings inoculated with *Ochrobactrum* sp. SSR. Rhizoscanning of wheat seedlings showed root length, diameter, and area of projection were improved with % increase ranging from 18 to 46%, 44–84% and 9–38%, respectively in response to PSB inoculation. Other parameters of root, i.e., root length (46–70 cm), root tips (44–75), root surface area (7–10 cm^2^), and area of projection (2–3 cm^2^) were observed to be significantly higher in PSB-inoculated seeds compared to uninoculated controls (Table 2).

**TABLE 2:** Effects of PSB on wheat germination, vigor index, and root morphology.

Treatments	Germination (%)	Vigor index	Root length (cm)	Root surface area (cm^2^)	Root diameter (mm)	Root volume (cm^3^)	Area of projection (cm^2^)	Number of root tips	Number of forks	No. of crossings
***Enterobacter* sp. ZW9**	91.00 ± 4.58 ABC	2111.00 ± 81.28 D	68.92 ± 3.85 A	10.16 ± 0.63 A	0.54 ± 0.03 C	0.19 ± 0.010 B	3.23 ± 0.19 AB	54.67 ± 3.06 B	149.67 ± 7.51 A	17.67 ± 0.76 A
***Enterobacter* sp. ZW32**	95.00 ± 5.00 AB	2093.33 ± 95.00 D	61.20 ± 3.73 B	9.31 ± 0.39 B	0.67 ± 0.03 B	0.19 ± 0.009 B	2.97 ± 0.13 B	53.00 ± 2.65 B	110 ± 5.57.00 B	11.67 ± 0.76 C
***Ochrobactrum* sp. SSR**	97.33 ± 4.51 A	2886.00 ± 68.17 A	69.92 ± 3.08 A	10.25 ± 0.43 A	0.77 ± 0.30 A	0.25 ± 0.013 A	3.26 ± 0.23 A	74.67 ± 2.52 A	116.67 ± 5.51 B	13.33 ± 0.76 B
***Enterobacter* sp. D1**	90.00 ± 4.58 BC	2453.67 ± 68.13 BC	45.79 ± 2.17 D	7.04 ± 0.32 D	0.41 ± 0.02 E	0.14 ± 0.007 D	2.24 ± 0.13 D	40.00 ± 2.00 D	64.00 ± 3.61 C	12.00 ± 0.50 C
***Pantoea* sp. S1**	91.33 ± 4.73 ABC	2527.33 ± 79.43 B	56.56 ± 2.80 B	9.76 ± 0.37 AB	0.47 ± 0.02 D	0.17 ± 0.009 C	3.11 ± 0.20 AB	48.67 ± 2.52 C	55.67 ± 2.52 DE	8.33 ± 0.52 D
***Bacillus* sp. TAYB**	88.00 ± 4.36 BCD	2355.00 ± 80.01 C	51.31 ± 2.54 C	7.97 ± 0.41 C	0.26 ± 0.01 F	0.09 ± 0.004 E	2.54 ± 0.14 C	51.33 ± 2.08 BC	62.33 ± 3.51 CD	8.33 ± 0.52 D
***Pseudomonas* sp. TJA**	84.33 ± 4.04 CD	1760.67 ± 72.01 E	49.48 ± 2.61 CD	7.99 ± 0.35 C	0.22 ± 0.01 F	0.13 ± 0.006 D	2.55 ± 0.08 C	43.67 ± 2.31 D	55.67 ± 2.52 DE	5.97 ± 0.38 E
**Uninoculated control**	81.33 ± 4.16 D	1304.00 ± 50.48 F	37.48 ± 1.54 E	6.39 ± 0.35 D	0.12 ± 0.01 G	0.07 ± 0.003 F	2.03 ± 0.05 D	26.00 ± 1.00 E	50.00 ± 2.65 E	4.67 ± 0.28 F

*Effect of PSB inoculation on root morphology parameters. Seeds were inoculated with PSB in water agar medium in a plate assay and incubated at room temperature for 7 days. All values are an average of six biological replicates. ± represents standard deviation. Means followed by the same letter differ non-significantly at p = 0.05 according to LSD.*

### Hydroponic Experiment to Study the Effect of PSB on Root Exudation and Anti-oxidant Enzymes

**FIGURE 4:**
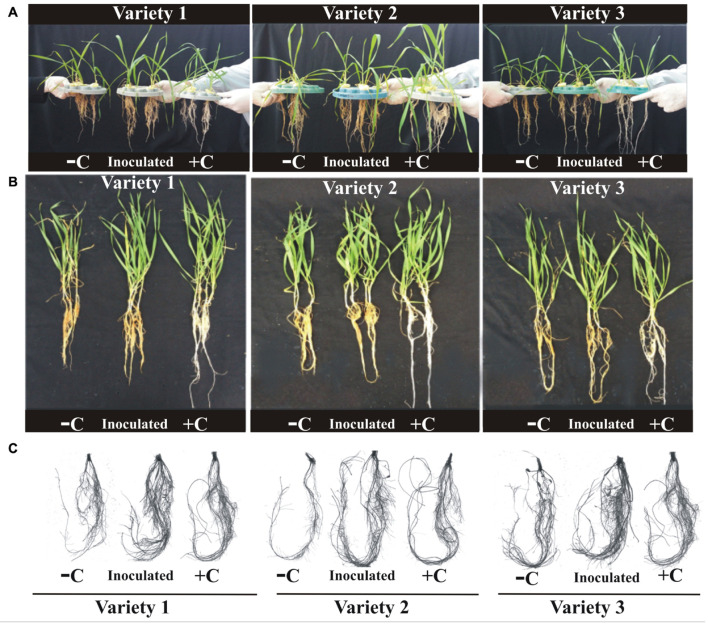
Effect of PSB consortia on growth of hydroponically grown different wheat varieties **(A,B)**. Rhizoscanning shows the root architecture **(C)**. Data were recorded at 45 days of cultivation in hydroponic system. Uninoculated seeds were used as controls. Plants in six biological replicates were used to analyze different root parameters. DPI: Days post-inoculation; PSB: Phosphate-solubilizing bacteria; +C: uninoculated positive control; –C: uninoculated negative control; Variety 1: Faisalabad-08, Variety 2: Fakhr-e-Sarhad, Variety 3: Benazir-13.

The effect of PSB on wheat root exudation and anti-oxidant production was studied in a hydroponic experiment in the greenhouse. Plants inoculated with selected PSB consortia had a positive impact on growth parameters of tested wheat varieties (variety 1, variety 2, and variety 3) compared to an uninoculated positive control (plants supplemented with Hoagland containing all nutrients including P) and uninoculated negative control (supplemented with modified Hoagland containing 1 g L^–1^ TCP as P-deficient solution; Figure 4). Inoculations (supplemented with modified Hoagland containing 1 g L^–1^ TCP as P-deficient solution) with respective bacterial consortia resulted in improved plant growth (shoot length and plant fresh and dry weight) and plant P content (1.3–1.6%) of three tested wheat varieties, compared to uninoculated controls (negative and positive; Supplementary Table S4).

Rhizoscanning of hydroponically grown wheat seedlings showed improved root architecture indicated by an increase in total root length (24–27%), root tips (46–53%), root surface area (29–35%), root volume (36–48%), and area of projection (28–47%) of three wheat varieties in response to inoculation with consortium-1, consortium-2, and consortium-3. Several other parameters of root, i.e., root length (458–549 cm), root tips (706–827), root surface area (54–58 cm^2^), and projection area (17–19 cm^2^) were observed to be significantly higher in seedlings inoculated with P-solubilizing bacteria when compared to uninoculated controls (Table 3).

**TABLE 3:** Effect of phosphate-solubilizing bacteria on root vigor index and morphological traits of hydroponically grown wheat varieties.

Wheat varieties	Treatments	Root length (cm)	Root surface area (cm^2^)	Root diameter (mm)	Root volume (cm^3^)	Area of projection (cm^2^)	Number of root tips	Number of forks	No. of crossings
**Variety 1**									
	**Negative control**	418 ± 20.07 CD	34.96 ± 1.87 C	0.318 ± 0.02 CD	0.25 ± 0.02 D	09.23 ± 0.88 B	387 ± 19.64 C	1934 ± 154 D	0.32 ± 0.02 CD
	**Consortium-1**	549 ± 25.45 AB	53.97 ± 2.75 A	0.371 ± 0.02 A	0.48 ± 0.01 AB	17.66 ± 0.47 A	827 ± 30.96 A	3207 ± 160 AB	0.48 ± 0.01 AB
	**Positive control**	557 ± 26.57A	52.22 ± 2.08 AB	0.358 ± 0.02 AB	0.41 ± 0.02 BC	17.15 ± 0.90 A	686 ± 33.43 AB	3129 ± 960 ABC	0.41 ± 0.02 BC
**Variety 2**									
	**Negative control**	341 ± 16.96 D	37.09 ± 1.63 C	0.313 ± 0.20 D	0.28 ± 0.02 D	11.81 ± 0.90 B	406 ± 20.50 C	2358 ± 118 BCD	0.26 ± 0.01 D
	**Consortium-2**	470 ± 24.99 ABC	58.65 ± 2.26 A	0.375 ± 0.20 A	0.55 ± 0.02 A	18.67 ± 0.53 A	795 ± 39.53 A	3828 ± 191 A	0.54 ± 0.03 A
	**Positive control**	502 ± 28.27 ABC	51.56 ± 2.03AB	0.347 ± 0.02 B	0.45 ± 0.03 AB	16.41 ± 0.87 A	687 ± 34.18 AB	3113 ± 960 ABC	0.45 ± 0.03 AB
**Variety 3**									
	**Negative control**	338 ± 18.98 D	40.00 ± 2.07 BC	0.334 ± 0.02 BC	0.32 ± 0.02 CD	12.73 ± 0.69 B	379 ± 19.97 C	2171 ± 108 CD	0.29 ± 0.02 D
	**Consortium-3**	458 ± 22.27 BC	56.03 ± 2.10 A	0.371 ± 0.02 A	0.50 ± 0.02 AB	18.35 ± 0.90 A	706 ± 35.22 AB	3036 ± 152 ABC	0.50 ± 0.02 AB
	**Positive control**	512 ± 31.40 ABC	53.61 ± 2.78 A	0.351 ± 0.02 AB	0.48 ± 0.02 AB	17.52 ± 0.86 A	590 ± 29.77 B	3001 ± 130 ABC	0.48 ± 0.03 AB

*Effect of PSB inoculations on root morphology parameters. Seeds were inoculated with PSB in Hoagland solution in hydroponics and harvested at the 45^th^ day. All values are an average of six biological replicates. ± represents standard deviation. Means followed by the same letter differ non-significantly at p = 0.05 according to LSD. Variety 1: Faisalabad-08, Variety 2: Fakhr-e-Sarhad, Variety 3: Benazir-13.*

Evaluation of consortium-1 strains (SSR, ZW9, and ZW32) on wheat variety1 at 45 DAS showed overall improved growth with increased shoot length (36 cm), fresh weight (2.6 g plant^–1^) and dry weight (0.9 g plant^–1^) as compared to uninoculated positive and negative controls. Moreover, consortium-1, consortium-2, and consortium-3 significantly increased plant P (1.3–1.6%) as compared to uninoculated positive and negative controls along with other growth parameters in wheat varieties 1, 2, and 3, respectively (Supplementary Table S4).

### Root Exudate Analysis

**FIGURE 5:**
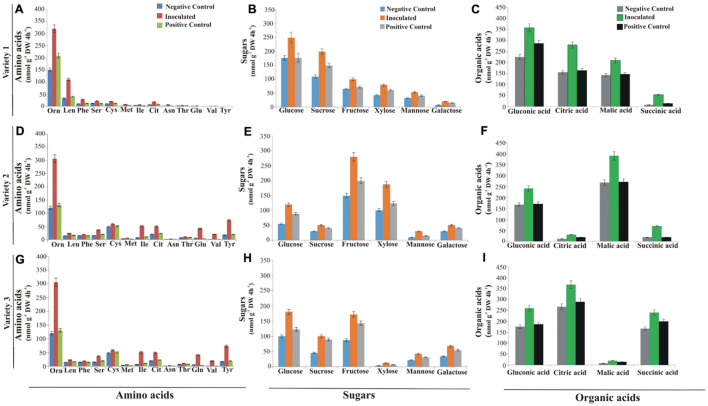
Differential root exudates of wheat varieties grown hydroponically. Root exudates of variety 1 amino acids **(A)**, sugars **(B)** and organic acids **(C)**. Root exudates of variety 2 amino acids **(D)**, sugars **(E)**, and organic acids **(F)**. Root exudates of variety 3 amino acids **(G)**, sugars **(H)**, and organic acids **(I)**. Root exudates were collected over a period of 4 h. Data are the mean of six biological replicates, while bars represent mean standard error; *n* = 4. Orn, Ornithine; Leu, Leucine; Phe, Phenylalanine; Ser, Serine; Cys, Cystine; Met, Methionine; Ile, Isoleucine; Cit, Citrulline; Asn, Asparagine; Thr, Threonine; Glu, Glutamine; Val, Valine; and Tyr, Tyrosine; Varieties 1, Faisalabad-08; Variety 2, Fakhr-e-Sarhad; Variety 3, Benazir-13; DW, dry weight.

Effect of PSB inoculations on wheat root exudation was analyzed hydroponically in greenhouse. Major root exudate components characterized and quantified by HPLC showed that root samples from PSB-inoculated plants of three wheat varieties produced a higher amount of amino acids, sugars, and organic acids as compared to positive and negative controls. Thirteen amino acids were released from the roots of each wheat variety with variable compositions. Ornithine was the most abundant amino acid (356 nmol g^–1^ DW 4 h^–1^) in inoculated roots of wheat variety 3 (Figure 5G) followed by 320 nmol g^–1^ DW 4 h^–1^ in inoculated roots of wheat variety 1 (Figure 5A) and 306 nmol g^–1^ DW 4 h^–1^ in inoculated roots of wheat variety 2 (Figure 5D) as compared to uninoculated negative and positive controls. Root samples from positive controls produced a higher amount of amino acids as compared to negative controls. Serine (63 nmol g^–1^ DW 4 h^–1^) and isoleucine (48 nmol g^–1^ DW 4 h^–1^) were the next most abundant amino acids, while citrulline, tyrosine, and valine were the least abundant amino acids released from roots of wheat variety 3. In the case of wheat variety 2, cysteine (59 nmol g^–1^ DW 4 h^–1^) was the second most abundant amino acid produced after ornithine, while asparagine and valine (2 nmol g^–1^ DW 4 h^–1^) were the least abundant amino acids. Leucine (110 nmol g^–1^ DW 4 h^–1^) was the most abundant, while valine and tyrosine were the least abundant amino acids produced by wheat variety 1.

Six sugars (glucose, sucrose, fructose, xylose, mannose, and galactose) were released in variable quantities from roots of all three wheat varieties. Glucose was the most abundantly produced sugar in inoculated plants of both variety 1 (250 nmol g^–1^ DW 4 h^–1^; Figure 5B) and variety 3 (180 nmol g^–1^ DW 4 h^–1^; Figure 5H), respectively, in comparison to negative and positive controls, while fructose was the most abundant (280 nmol g^–1^ DW 4 h^–1^) and mannose was the least abundant (30 nmol g^–1^ DW 4 h^–1^) sugar produced in inoculated treatments of variety 2 as compared to uninoculated controls (Figure 5E).

Organic acids (gluconic acid, malic acid, citric acid, and succinic acid) were detected in root exudates of all wheat varieties in variable composition, where quantities of organic acids were higher in inoculated treatments. The most abundant organic acids produced in exudates of variety 1 were gluconic acid (358 nmol g^–1^ DW 4 h^–1^; Figure 5C), followed by citric acid (280 nmol g^–1^ DW 4 h^–1^), malic acid (210 nmol g^–1^ DW 4 h^–1^), and succinic acid (54 nmol g^–1^ DW 4 h^–1^) in variety 1 as compared to uninoculated negative and positive controls; whereas malic acid (388 nmol g^–1^ DW 4 h^–1^) and citric acids (368 nmol g^–1^ DW 4 h^–1^) were most abundantly produced in root exudates of inoculated treatments of variety 2 and variety 3, respectively, compared to uninoculated negative and positive controls (Figures 5F,I).

**FIGURE 6:**
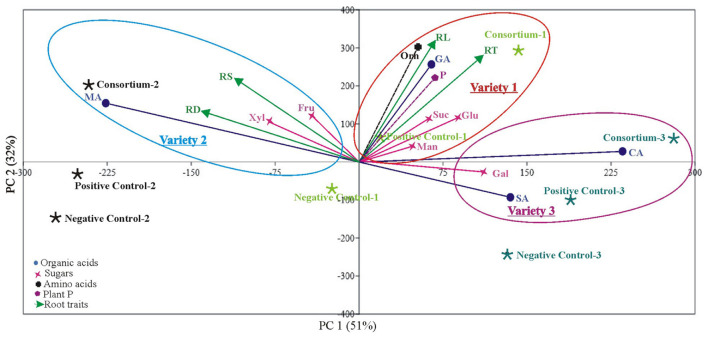
Principal component analysis of root parameters (root exudation and root architecture) and plant P across different wheat varieties in response to PSB inoculation. The first two components explain most of the variation found in the dataset (PC1 and PC2) in wheat variety 1, variety 2, and variety 3. The points represent mean values of each combination of plant P, root exudates, root morphological parameters in correlation to different treatments (inoculation, positive control, and negative control). Loadings (arrows, circles, star, and boxes) indicate directions and strength of parameters in the dataset. RT: Root tips, RD: Root diameter, RS, Root surface area; RL, Root length; GA, Gluconic acid; MA, Malic acid; CA, Citric acid; SA, Succinic acid; Fru, Fructose; Gal, Galactose; Glu, Glucose; Man, Mannose; Suc, Sucrose; Xyl, Xylose and P; Plant phosphorous. Variety 1, Faisalabad-08; Variety 2, Fakhr-e-Sarhad; Variety 3, Benazir-13.

Principal component analysis (PCA) showed clustering of plant P content, root morphological traits (root surface area, root diameter, root length, and root tips), and root exudations (sugars, amino acids, and organic acids) of PSB-inoculated plants in one group indicating a positive correlation among these parameters (Figure 6).

### Antioxidant Enzyme Analysis

**FIGURE 7:**
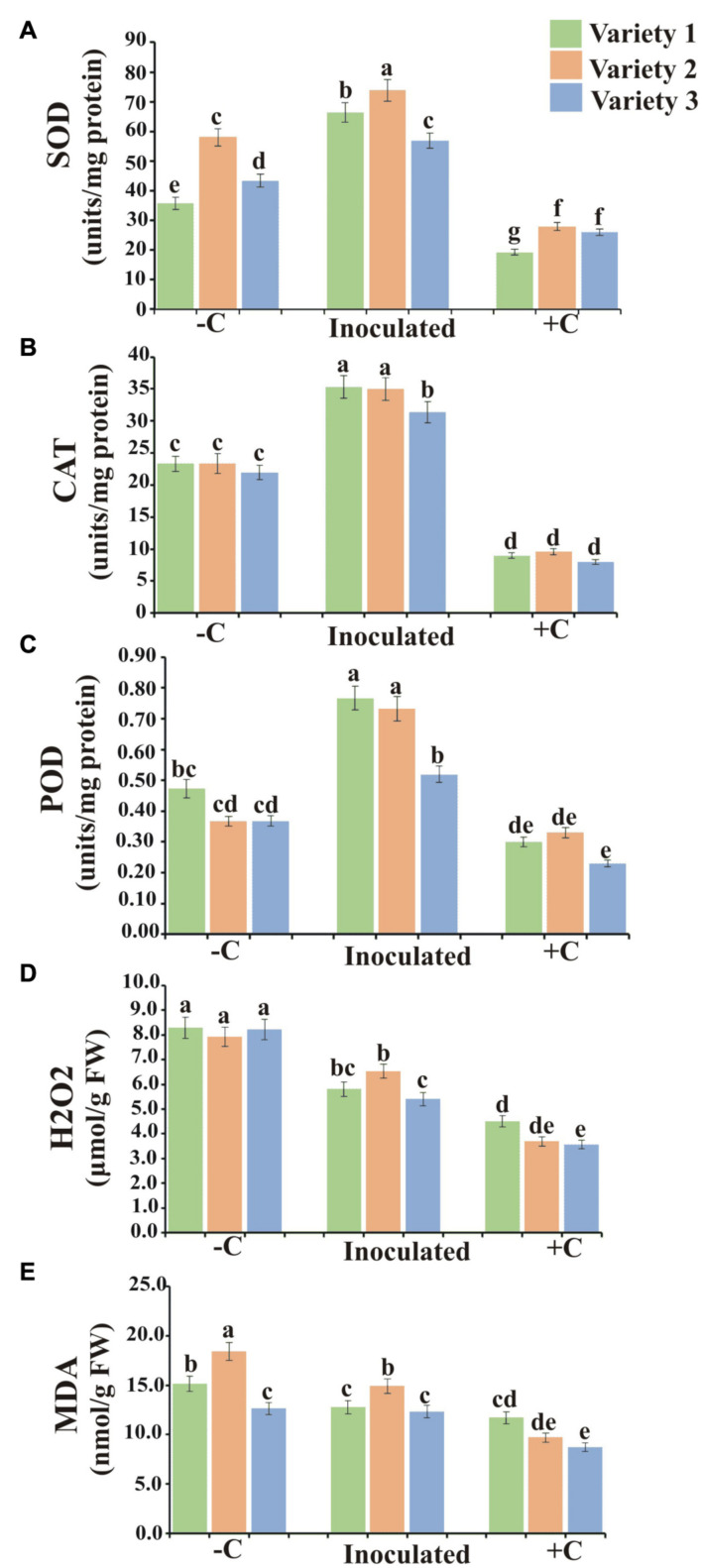
Anti-oxidant enzyme activity in hydroponically grown wheat varieties. Superoxide dismutase **(A)**, Catalase **(B)**, Peroxidase **(C)**, H_2_O_2_ **(D)**, and MDA contents **(E)** in plants of wheat varieties 1, 2, and 3 at harvest. Values are the average of six biological replicates. Bars show standard error of means. Significant difference (*p <* 0.05) among treatments is represented by different letters (a, b, c). Variety 1, Faisalabad-08; Variety 2, Fakhr-e-Sarhad; Variety 3, Benazir-13; +C, uninoculated positive control; –C, uninoculated negative control.

Accumulated ROS-induced oxidative damage and MDA content in plant roots were reduced by PSB inoculations. All wheat varieties showed higher H_2_O_2_ contents in negative controls as a result of induced P deficiency. The production of super dismutase (SOD), catalase (CAT), and peroxidase (POD) in PSB-inoculated treatments of all wheat varieties were higher as compared to negative and positive controls. In addition, CAT, SOD, and POD were the highest produced enzymes in wheat variety 2 followed by variety 1 and variety 3 (Figure 7).

### Pot Experiment Using Soils of Different Agro-Ecological Zones

Effect of soil-specific consortia on recommended wheat varieties of three major wheat-growing provinces was studied in a pot experiment, nine soils (site nos. 1–9, Supplementary Table S5) of province-1, three soils (site nos. 1–3, Supplementary Table S5) of province-2, and four soils (site no. 1–4, Supplementary Table S5) of province-3 under controlled conditions at NIBGE, Faisalabad. At 45 DAS, plant growth-promoting parameters showed that selected PSB had a positive impact on wheat growth parameters in all soils treated with their respective consortium compared to respective uninoculated controls (Table 4).

**TABLE 4:** Effect of phosphate-solubilizing bacteria on growth parameters of wheat varieties grown in soils of different agro-ecological zones.

Treatments		Soil sites	Fresh weight (g plant^–1^)	Dry weight (g plant^–1^)	Shoot length (cm)	Root length (cm)	Plant P content (%)
**Province1**							
	**Inoculated (IN)**	**Site 1**	2.13 ± 0.10 DEFG	0.72 ± 0.02 CDEF	23.67 ± 1.10 DEFG	26.00 ± 1.30 BC	1.23 ± 0.04 E
		**Site 2**	3.14 ± 0.20 BC	0.89 ± 0.17 ABC	26.47 ± 1.30 C	27.33 ± 1.50 AB	1.91 ± 0.06 C
		**Site 3**	3.48 ± 1.00 AB	0.92 ± 0.20 AB	28.67 ± 1.50 B	27.50 ± 1.50 B	2.07 ± 0.10 B
		**Site 4**	2.30 ± 0.10 DEF	0.73 ± 0.10 DEFG	24.83 ± 1.70 CDE	25.33 ± 1.50 C	1.16 ± 0.05 EF
		**Site 5**	2.62 ± 0.1 CDE	0.77 ± 0.10 BCDE	25.17 ± 1.00 CDE	21.33 ± 1.00 D	1.13 ± 0.04 EF
		**Site 6**	2.35 ± 0.10 DEF	0.78 ± 0.02 BCD	25.33 ± 1.50 CD	26.2 ± 1.00 BC	1.66 ± 0.05 D
		**Site 7**	3.89 ± 0.10 A	1.05 ± 0.10 A	32.00 ± 0.50 A	29.50 ± 1.20 A	4.63 ± 0.20 A
		**Site 8**	2.92 ± 0.20 BCD	0.87 ± 0.04 ABC	25.50 ± 1.50 CD	26.17 ± 1.00 BC	1.82 ± 0.20 C
		**Site 9**	2.13 ± 0.2 DEFG	0.66 ± 0.10 DEFG	24.00 ± 1.30 DEF	21.67 ± 1.80 D	1.13 ± 0.06 EF
	**Uninoculated Controls (UC)**	**Site 1**	1.29 ± 0.01 HI	0.43 ± 0.04 HI	21.83 ± 1.00 GHI	18.07 ± 0.50 EF	0.72 ± 0.03 GHI
		**Site 2**	1.78 ± 0.02 FGH	0.55 ± 0.04 FGH	21.50 ± 1.30 HI	14.17 ± 0.80 H	0.44 ± 0.03 J
		**Site 3**	2.01 ± 0.50 EFGH	0.46 ± 0.10 GHI	23.17 ± 1.60 EFGH	21.16 ± 1.60 DEF	1.12 ± 0.03 EF
		**Site 4**	1.85 ± 0.05 EFGH	0.32 ± 0.05 I	18.67 ± 0.50 K	19.50 ± 0.30 EF	0.75 ± 0.02 GH
		**Site 5**	1.34 ± 0.04 HI	0.57 ± 0.01 EFGH	21.00 ± 1.00 IJ	15.33 ± 0.06 GH	0.62 ± 0.03 HI
		**Site 6**	1.04 ± 0.03 I	0.42 ± 0.03 HI	21.00 ± 1.00 IJ	20.67 ± 1.50 DEF	0.76 ± 0.06 G
		**Site 7**	2.10 ± 0.10 DEFG	0.37 ± 0.03 HI	17.67 ± 1.00 K	18.07 ± 1.00 FG	0.44 ± 0.03 J
		**Site 8**	0.98 ± 0.10 GHI	0.33 ± 0.03 HI	19.17 ± 1.20 JK	19.53 ± 0.60 EF	0.59 ± 0.59 I
		**Site 9**	1.90 ± 0.50 EFGH	0.43 ± 0.03 HI	22.00 ± 2.00 FGHI	21.00 ± 1.20 DEF	1.04 ± 0.03F
**Province 2**							
	**IN**	**Site 1**	4.14 ± 0.21 A	0.91 ± 0.02 A	27.50 ± 1.30 A	27.47 ± 2.0 A	1.36 ± 0.01 A
		**Site 2**	1.80 ± 0.06 B	0.62 ± 0.02 B	24.10 ± 1.15 B	26.83 ± 1.7 A	1.19 ± 0.05 B
		**Site 3**	1.68 ± 0.02 BC	0.43 ± 0.03 C	23.00 ± 1.00 B	23.13 ± 1.8 B	1.04 ± 0.05 C
	**UC**	**Site 1**	1.13 ± 0.06 D	0.42 ± 0.03 D	23.00 ± 1.00 B	21.83 ± 2.0 C	1.03 ± 0.01 C
		**Site 2**	1.41 ± 0.05 CD	0.33 ± 0.03 C	19.67 ± 0.76 C	16.67 ± 1.6 B	0.45 ± 0.04 E
		**Site 3**	1.13 ± 0.06 D	0.24 ± 0.03 E	18.83 ± 0.76 C	17.90 ± 1.0 C	0.95 ± 0.05 D
**Province 3**							
	**IN**	**Site 1**	1.59 ± 0.10 B	0.71 ± 0.01 B	26.00 ± 0.60 B	17.67 ± 0.60 D	1.16 ± 0.03 C
		**Site 2**	1.53 ± 0.10 B	0.46 ± 0.01 C	25.33 ± 1.50 B	20.00 ± 1.50 C	1.16 ± 0.05 C
		**Site 3**	1.89 ± 0.30 A	0.80 ± 0.02 A	29.00 ± 1.00 A	33.00 ± 1.00 A	2.26 ± 0.15 A
		**Site 4**	1.96 ± 0.03 A	0.77 ± 0.01 A	31.00 ± 1.00 A	29.00 ± 1.00 B	1.39 ± 0.10 B
	**UC**	**Site 1**	0.75 ± 0.20 D	0.35 ± 0.03 D	22.67 ± 0.80 C	14.67 ± 0.80 F	1.01 ± 0.02 D
		**Site 2**	1.10 ± 0.10 C	0.35 ± 0.03 D	20.00 ± 0.90 D	15.67 ± 0.60 EF	1.13 ± 0.06 CD
		**Site 3**	1.10 ± 0.10 C	0.30 ± 0.03 D	19.00 ± 1.20 D	17.00 ± 1.00 DE	0.64 ± 0.04 E
		**Site 4**	0.62 ± 0.10 D	0.14 ± 0.03 E	19.37 ± 0.80 D	16.83 ± 0.80 DE	0.52 ± 0.03 E

*Effect of bacterial inoculation on various wheat growth parameters at 45 days after sowing in pot experiment under greenhouse conditions Values are an average of 6 biological replicates. ± Represent the standard deviations (SD).*

*Means with significant difference (P < 0.05) among treatments of each province is represented by different letter.*

*For **Province 1;** Site1: Dera Ghazi Khan, Site2: Faisalabad, Site3: Jhang, Site4: Rahim Yar Khan, Site5: Multan, Site6: Sheikhupura, Site7: Gujranwala, Site8: Sialkot, Site9: Rawalpindi. For **Province 2;** Site1: Dir, Site2: Swat, Site3: Peshawar and **Province 3;** Site1: Hyderabad, Site 2: Tandojam, Site3: Sanghar and Site4: Larkana. Province 1: Punjab, Province 2: Khyber Pakhtunkhwa and Province 3: Sindh. IN, Inoculated; UN, Un-inoculated Control.*

The plant growth of wheat variety 1 inoculated with consortium-1 was significantly increased. Significant increase in plant shoot length (32 cm), root length (29.5 cm), and plant dry weight (1.05 g) of consortium-1-inoculated wheat (variety-1) were observed in soils of site 7 (Gujranwala) with 40% increase in root length as compared to uninoculated controls. Increased shoot length (28 cm), root length (27 cm), and dry weight (0.91 g) were observed in inoculated (consortium-2) wheat variety 2 grown in site 1 (Dir) soil of Province 2 with 21% increased root length as compared to uninoculated controls; whereas inoculation of plants (variety 3) with consortium-3 improved shoot length (29 cm), root length (33 cm), and plant dry weight (0.80 g) with a 48% increase in root length as compared to uninoculated controls in site 3 soil (Sanghar) of Province 3 (Table 4).

**FIGURE 8:**
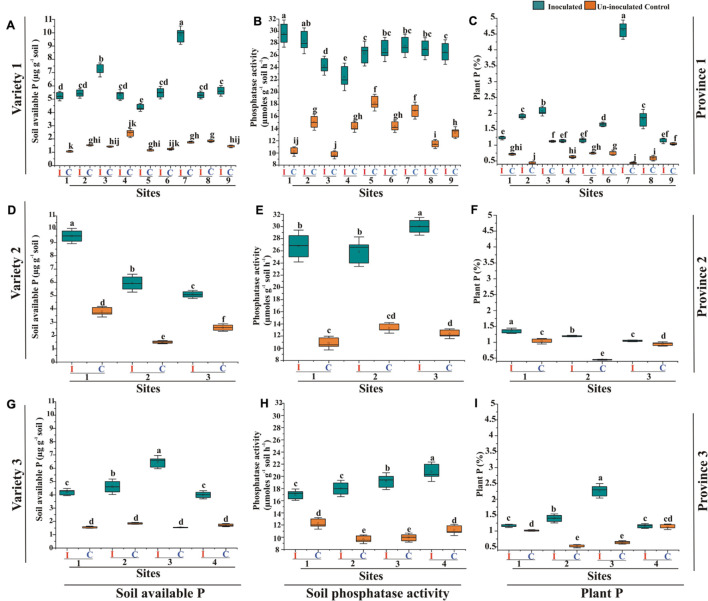
Effect of PSB inoculation on soil and plant P parameters of wheat varieties grown in soils from different agro-ecological zones compared to uninoculated controls. Evaluation of consortium-1 for soil available P **(A)**, soil phosphatase activity **(B)**, and plant P content **(C)** of wheat variety-1 grown in soils of province-1. Evaluation of PSB consortium-2 for soil available P **(D)**, soil phosphatase activity **(E)**, and plant P content **(F)** of wheat variety-2 grown in soils of province-2. Evaluation of consortium-3 for soil available P **(G)**, soil phosphatase activity **(H)**, and plant P content **(I)** of wheat variety-3 in soils of province-3. Different small (a, b, c) letters above each box indicate significantly differences between the treatments according at *p* < 0.05. I: Inoculated, C: Uninoculated control. For **Province 1**: Site 1: Dera Ghazi Khan, Site 2: Faisalabad, Site 3: Jhang, Site 4: Rahim Yar Khan, Site 5: Multan, Site 6: Sheikhupura, Site 7: Gujranwala, Site 8: Sialkot, Site 9: Rawalpindi. For **Province 2:** Site1: Dir, Site 2: Swat, Site 3: Peshawar. For **Province 3:** Site 1: Hyderabad, Site 2: Tandojam, Site 3: Sanghar, and Site 4: Larkana. Province-1: Punjab, Province-2: Khyber Pakhtunkhwa, and Province-3: Sindh; Variety 1: Faisalabad-08, Variety 2: Fakhr-e-Sarhad, Variety 3: Benazir-13.

Consortium-1, 2, and 3 significantly increased (up to 4%) plant P content in respective wheat varieties grown in soils of site 7 of province-1, site 1 of province-2, and site 3 of province -3 as compared to uninoculated plants grown in soils of respective sites (Figure 8). Soils of province 1 and province 3 are majorly comprised of irrigated plains and soils of province 2 prevalent with dry mountains. The inoculated plants with consortium-1 increased soil available P (5.2–9.8 μg g^–1^ soil) and phosphatase activity (24.3–29.6 μmol g^–1^ soil h^–1^) in inoculated soils of province-1. Consortium-2 increased soil available P up to 5–9 μg g^–1^ soil and phosphatase activity 25–30 μmol g^–1^ soil h^–1^. Consortium-3 also increased available P up to 4-6.5 μg g^–1^ soil and phosphatase activity (17–20 μmol g^–1^ soil h^–1^; Figure 8 and Supplementary Table S6).

A positive correlation was found as a result of inoculation of three PSB consortia on plant growth (plant fresh and dry weight, root-shoot length, and plant P), soil available P, and soil phosphatase activity for recommended wheat varieties grown in native soils of Province 1, Province 2, and Province 3 (Supplementary Figure S2).

### Evaluation of PSB Consortium for Wheat Yield Parameters

P-solubilizing consortia significantly enhanced wheat growth in a pot experiment under net house conditions. PSB consortium-1 inoculated wheat variety1 showed increased grain yield (5.95 g plant^–1^) in comparison to 80% uninoculated control (80% of recommended DAP dose). A significant increase (4.1%) in seed P was observed in inoculated plants compared to 80% uninoculated control. An increase in soil available P (6.4 μg g^–1^ soil) and phosphatase activity (24 μmol g^–1^ soil h^–1^) was observed with PSB inoculation (Table 5). A positive correlation was found between wheat yield parameters, i.e., seed P content, wheat grain yield, soil available P, and gluconic acid produced by P-solubilizing bacteria in an *in vitro* quantification assay (Supplementary Figure S3).

**TABLE 5:** Effect of phosphate-solubilizing bacteria on plant growth, soil P contents, and yield of wheat in a pot experiment under net house conditions.

Treatments	35 DAS^1^							120 DAS^2^						
	Plant weight	Shoot length (cm)	Root length (cm)	Plant P content	Soil available P	Phosphatase	Viable	No. of tillers (tillers plant^–1^)	Shoot length (cm)	Plant biomass	Grain yield (g plant^–1^)	Seed P content	Soil available P	Phosphatase
	(g plant^–1^)			(%)	(mg g^–1^ soil)	activity	(CFU g-^1^soil)			(g plant^–1^)		(%)	(mg g^–1^ soil)	activity
						(mmol g^–1^ soil h^–1^)								(mmol g^–1^ soil h^–1^)
**Cons-1 (80%)**	1.84 ± 0.09 A	35.33 ± 1.77 A	11.33 ± 0.58 A	4.56 ± 0.23 A	6.30 ± 0.31 A	11.47 ± 0.57 A	9.68x10^7^	10.66 ± 0.55 A	78.20 ± 3.90 A	10.01 ± 0.51 A	5.95 ± 0.31 A	4.13 ± 0.21 A	6.42 ± 0.32 A	24.10 ± 1.21 A
**Control 80%**	0.52 ± 0.03 C	19.67 ± 0.99 B	06.73 ± 0.34 C	1.00 ± 0.05 C	0.87 ± 0.04 B	6.60 ± 0.33 B	2.58x10^7^	4.67 ± 0.23 C	71.43 ± 3.54 A	3.97 ± 0.24 C	2.44 ± 0.13 B	2.20 ± 0.11 C	1.22 ± 0.06 B	13.23 ± 0.96 C
**Control 100%**	0.81 ± 0.04 B	19.97 ± 1.00 B	08.67 ± 0.42 B	2.10 ± 0.11 B	1.24 ± 0.06 B	6.87 ± 0.34 B	4.34x10^7^	8.83 ± 0.44 B	77.78 ± 3.90 A	7.74 ± 0.40 B	5.47 ± 0.26 A	3.54 ± 0.18 B	1.36 ± 0.07 B	15.92 ± 0.81 B

*^1,2^ Effect of bacterial inoculation on wheat growth and yield parameters, P uptake and soil nutrients at 35 days after sowing (DAS) and at 120 DAS in a pot experiment at Faisalabad grown wheat during winter season 2018–19. Control 80% and Control 100% represent non-inoculated controls supplemented with 80% of the recommended dose of P fertilizer and full recommended dose of P fertilizer, respectively. PSB (1 × 10^9^ CFU ml^–1^) were seed-inoculated before sowing. Inoculated pots were supplemented with 80% of recommended dose of DAP fertilizer. Means are the average of six biological replicates arranged in CRD. Means followed by the same letter differ non-significantly at p = 0.05 according to LSD. ± Represents the standard deviations (SD). Six plants were grown in earthen pots (diameter: 12 in, height: 14 in) containing 12 kg soil.*

### Detection of Inoculated PSB

**FIGURE 9:**
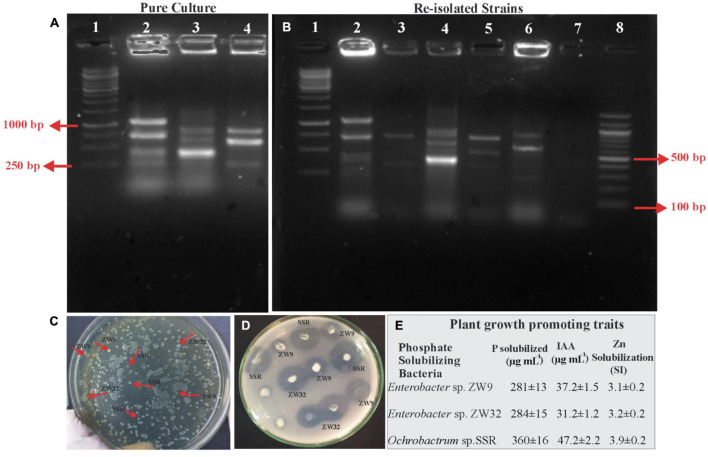
Re-isolation of inoculated PSB colonies. Photograph of gel indicates BOX PCR patterns of pure culture of PSB from wheat rhizosphere, 1: 1Kb DNA ladder; 2: SSR, 3: ZW32, 4: ZW9 **(A)**. Photograph of gel indicates re-isolated colonies of PSB that are similar morphologically to the inoculated consortium-1 PSB. 1: 1Kb DNA ladder, 2: Re-isolated SSR colony, 3: Non-specific colony, 4: Re-isolated ZW32 colony, 5: Re-isolated ZW9, 6: Non-specific colony, 7: -ve Control; 8: 100 bp DNA ladder **(B)**. Plate showing re-isolated colonies of SSR, ZW32, and ZW9 **(C)**. NBRIP Plate showing solubilization zones formed by re-isolated colonies of SSR, ZW32, and ZW9 **(D)**. Plant growth-promoting attributes of re-isolated PSB strains for confirmation of inoculated PSB: P solubilization and IAA production were detected quantitatively by HPLC, and Zinc solubilization was confirmed by formation of halo zone on agar plate added with ZnO **(E)**. Values are an average of six biological replicates. *Enterobacter* spp. ZW9, ZW32, *Ochrobactrum* sp. SSR, -ve control represents negative control.

To confirm the presence of inoculated PSB in the rhizosphere of wheat variety-1 grown in a pot experiment under net house conditions, bacterial population was recorded using viable count at 35 DAS. The re-isolated colonies of *Enterobacter* spp. ZW9, ZW32, and *Ochrobactrum* sp. SSR were identified based on the morphological characteristics and other plant growth-promoting attributes, i.e., P solubilization (224–345 μg ml^–1^, IAA production (4–47 μg ml^–1^), and zinc solubilization (SI: 2.9–3.9) in comparison to pure cultures. BOX-PCR confirmed the inoculated P-solubilizing bacteria. BOX fingerprinting of re-isolated colonies were found identical to those of pure cultures (Figure 9).

## Discussion

Due to the depleting reservoirs of available P and high costs of phosphatic fertilizers, PSB-based biostimulants serve as an alternative and sustainable strategy in modern agriculture for food and environmental security. Therefore, the present study was designed to get comprehensive data of wheat rhizosphere-associated PSB from different soils belonging to major wheat-growing agro-ecological zones of Pakistan (situated between 24 and 37 north and longitude of 61 to 75 east; Supplementary Table S1 and Supplementary Figure S1). We determined the beneficial effects of native PSB inoculum on wheat growth, yield, and P uptake. Previous studies reported the presence of PSB in the soils of selected areas of Pakistan (Suleman et al., 2018), but major wheat growing agro-ecological zones remained unexplored.

The ecological approach developed in this study enabled us to select 25 potential PSB. Efficient P solubilizers belonged to the genus *Enterobacter* (strains: ZW9, ZW32 and D1), *Ochrobactrum* (SSR), *Pantoea* (S1), and *Pseudomonas* (TJA). Increase in soluble P concentration was observed in quantitative assay with a concomitant decrease in pH up to 3.6 from initial pH 7 in liquid medium (Figure 2). We observed that pH of the medium decreased as soluble P increased. This might be because P-solubilizing bacteria can secrete organic acids in a culture medium to degrade TCP (insoluble P source) in order to increase soluble P (Penn and Camberato, 2019; Chouyia et al., 2020). Among organic acids detected by HPLC acetic acid, gluconic and citric acids were predominantly produced by selected P-solubilizing bacteria (Table 1). Three most efficient PSB strains, *Ochrobactrum* sp. SSR and *Enterobacter* spp. (ZW9, ZW32) produced higher gluconic acid and acetic acid. This indicates that apart from gluconic acid, other acids like acetic acid and citric acid are produced in significant levels by bacteria. Suleman et al. (2018) reported gluconic acid and acetic acid as dominantly produced acids by *Pseudomonas* sp. and *Enterobacter* sp., respectively. Differential production of these organic acids by PSB seems a key factor responsible for phosphate solubilization (Azaroual et al., 2020; Tian et al., 2021).

In order to link bacterial genetic potential with P-solubilizing activity, glucose dehydrogenase (*gcd*), *pqqE*, and phytase (*phy*) genes (Perez et al., 2007; Han et al., 2008; Chen et al., 2016) related to the organic and inorganic P solubilization were studied in selected PSB. *Gcd* was amplified in *Pantoea* sp. S1, while *phy* genes were amplified in both S1and *Enterobacter* sp. ZW32. The current study reported amplification of *PqqE* gene in ZW32 and SSR (Figure 3 and Supplementary Table S3). In our previous study (Rasul et al., 2021), expression analysis of multiple genes related to P-release pathways of PSB was carried out using qPCR. This study revealed that *Ochrobactrum* sp. SSR having *gcd* and *phy* genes can solubilize different organic and inorganic P sources (phytate, rock phosphate, and K_2_HPO_4_). SSR was found with multifaceted nature; hence, it was selected as a common P-solubilizing strain in consortia development. Efficient and compatible PSB (*Enterobacter* spp. ZW9, ZW32 and D1, *Ochrobactrum* sp. SSR, *Pantoea* sp. S1, *Pseudomonas* sp. TJA, and *Bacillus* sp. TAYB) with multiple plant growth-promoting (PGP) traits (i.e., IAA, siderophores, and zinc solubilization) was used to develop three consortia for their native soils and respective recommended wheat varieties. This is due to indigenous microbes that are most likely to promote plant growth as they are more adaptive with higher persistence in native soil (Glick, 2012; Souza et al., 2015).

Root exudation is an important process that regulates the interaction between root and soil microbes (Mommer et al., 2016). These metabolites are difficult to collect from real soil environments due to their chemical interaction with soil matrix and background microbial exudation (Olanrewaju et al., 2019; Kumar and Dubey, 2020; Pang et al., 2021). Therefore, in the present study, the hydroponic system was used to evaluate the effect of PSB consortia on root exudation with consequent changes in root architecture. Characterization and HPLC quantification of major root exudates from hydroponically grown wheat varieties showed that PSB-inoculated roots produced higher amounts of amino acids, organic acids, and sugars under P-deficient conditions as compared to uninoculated controls (Figure 5). It has been known that soil microbes can release certain compounds to stimulate the root exudation of primary metabolites (i.e., amino acids, organic acids, and sugars) that are passively lost from roots and utilized by rhizosphere-dwelling microbes (Canarini et al., 2019).

In the present study, 13 amino acids and six sugars were released from roots of three wheat varieties with variable compositions. Among amino acids, ornithine was abundantly found in root exudation of PSB-inoculated plants of three wheat varieties as compared to uninoculated plants; cumulative release of sugars in root exudates was higher in variety 1 and variety 2 compared to variety 3 (Figure 5). Amino acids in root exudates besides sugars tailor communities because they represent carbon and nitrogen substrates for microbial growth (Pongrac et al., 2020). Higher release of these exudates contributes to increase in bacterial persistence in the root rhizosphere (Nwachukwu et al., 2021). In return, bacteria can benefit plants by enhanced the nutrient absorbing capability of roots especially to facilitate phosphorus uptake (Grover et al., 2021). Furthermore, HPLC analysis revealed a significant increase of organic acids specifically gluconic acid (20–39%), malic acid (30%), and citric acid (21%–41%) released in exudates collected from PSB-inoculated three wheat varieties as compared to their respective uninoculated controls (Figure 5). Increased concentrations of oxalic, malic and citric acids in root exudates act as chemo-attractants for soil microbes during beneficial interactions (Pascale et al., 2020; Tsai et al., 2020).

Root response to P plays a major role in the modulation of root architecture as compared to other nutrients (Peris et al., 2020). Therefore, in addition, to study the root exudates, rhizoscanning of hydroponically grown plants was also carried out to explore the effect of PSB on root architecture. PSB inoculation modified root architecture, as indicated by an increase in total root length (24–27%), root tips (46–53%), root surface area (29–35%), root volume (36–48%), and area of projection (28–47%) of three wheat varieties provided with an insoluble source of P (TCP) as compared to uninoculated control. Significant increases in root diameter, root tips, and root surface area were key parameters modified in response to PSB inoculation (Table 3).

The root tip is the first part of the plant to explore a new soil environment and plays a crucial role in response to environmental stimiuli. A growing number of studies showed root tip functions as a control center for sensing externalnutrient concentration and translating it into an alteration of root architecture (Huang and Zhang, 2020; Grover et al., 2021). Increased root diameter promotes plant growth and development since thicker roots easily penetrate the soil, thereby anchoring the plant more effectively leading to efficient nutrient uptake (Freschet et al., 2020). In addition to improved architecture, a significant increase in growth and plant P contents of three wheat varieties was observed in plants inoculated with PSB consortia as compared to the uninoculated controls (Supplementary Table S4). Principal component analysis showed clustering of P content, root morphological traits, and root exudations of PSB-inoculated plants in one group, indicating a positive correlation between these parameters (Figure 6). PSB-stimulated primary metabolites may serve important functions in the soils as a key nutrient source for rhizosphere microbes and influence root-microbe relations (Pang et al., 2021).

Besides the effect of PSB on root architecture and exudation in hydroponics, these bacteria sustained the plant growth under P stress as indicated by a significant increase in plant antioxidant enzyme (SOD, POD, MDA, and CAT) activity with a concomitant decrease in MDA and H_2_O_2_ contents (Figure 7). There is increasing evidence that PGPR confers tolerance in plants to various abiotic stresses (Mokrani et al., 2020; Bhat et al., 2020; Khan et al., 2020; Camaille et al., 2021) by activating enzymes like CAT, POD, and SOD activity which act as primary reactive oxygen species (ROS) scavengers (Grover et al., 2021). These enzymes catalyze the O^2–^ to H_2_O_2_, and H_2_O_2_ is further converted into H_2_O and O_2_ by peroxidases, thereby enhancing tolerance and eliciting resistance in plants against oxidative stress (Li et al., 2020; Eljebbawi et al., 2021).

P-solubilizing efficacy of three developed PSB consortia was further subjected to *in planta* evaluation under natural soil conditions using soils collected from different agro-ecological zones of three provinces with their recommended wheat varieties. Results showed a significant increase (up to 4%) in plant P contents and plant root length (up to 48%) in soils of province-1 and province-3 majorly comprising irrigated plains and soils of province 2 prevalent in dry mountains in response to inoculation of soil-specific consortia (Supplementary Table S1 and Supplementary Figure S1). A significant increase of soil available P and phosphatase activity was observed in PSB-inoculated soils as compared to uninoculated controls (Figure 8). This is in line with Chen et al., 2021, who reported an elevated amount of available P in wheat-grown soil inoculated with PSB as compared to uninoculated control. Boubekri et al. (2021) reported the highest level of available P and greater stimulation of wheat plant growth in soils co-inoculated with phosphate-solubilizing consortium. Lack of region/soil-specific strains is one of the major constraints in the inconsistent performance of bio-inoculants. In this study, we tried to address this problem by developing region/soil-specific inoculum with expected outcome across wheat-cultivated agricultural lands. The use of region-specific strains ensures the optimal use of biofertilizers with expected outcomes over vast areas of agricultural lands (Mitter et al., 2021). PCA showed a significant positive correlation between all plant growth parameters (root length, plant P, soil available P, and soil phosphatase activity) in PSB-inoculated wheat varieties as compared to their respective uninoculated plants (Supplementary Figure S2). The increased P availability in the rhizosphere soil and its better aboveground translocation occurred either directly or indirectly in response to PSB inoculation results in better plant growth (Elhaissoufi et al., 2020).

Furthermore, to study the contribution of efficient PSB consortium-1 to increase the crop yield, a pot experiment was carried out using wheat variety 1 under net house conditions at NIBGE. Inoculated plants provided with 20% reduced DAP application showed a significant increase (7%) in grain yield, plant biomass (20%), seed P content with an increase in soil phosphatase activity, and subsequent availability of soil P (Table 5). Regression analysis confirmed a positive correlation between wheat yield parameters, i.e., seed P content, wheat grain yield, soil available P, and gluconic acid produced by P-solubilizing bacteria in an *in vitro* quantification assay (Supplementary Figure S3). Higher seed P content might be due to translocation of P to seed as previously reported by Feng et al. (2021). Enhanced soil phosphatase activity in PSB-inoculated treatments showed the ability of these PSB to convert organic P complexes in soil into an available form for plant uptake (Chawngthu et al., 2020; Chen et al., 2021). Previous studies demonstrated cost-effective use of PSB inoculations to increase grain yield and plant P in wheat, rice, and sugarcane along with reduced DAP dose (Suleman et al., 2018; Rasul et al., 2019; Rosa et al., 2020). These studies indicated that P-solubilizing microorganisms showed the best effect with reduced application of DAP fertilizers. The survival of inoculated strains of consortium-1 in root rhizosphere as studied by viable count and BOX-PCR revealed that these were rhizosphere-competent phosphobacteria. Moreover, plant growth-promoting traits of re-isolated bacteria in comparison with those of the pure culture of inoculated strain indicated the persistence of inoculated PSB (Figure 9).

The ecological approach used in the study allowed us to isolate and select multifaceted phosphate-solubilizing bacteria from different unexplored agro-ecological zones. These promising strains (ZW9, ZW32, and SSR), deposited to the DSMZ German culture collection (accession no. 109592-93 and 109610), serve as a global valuable pool. The phosphate solubilization activity of these potential PSB was highly promising in different types of soils and wheat varieties as statistically validated by improved plant growth, yield, and soil fertility-related traits like soil available P and soil phosphatase activity. This has lain a solid foundation for further utilization of these effective PSB candidates as soil/genotype-specific biofertilizers for wheat under a wide range of agro-ecological conditions intended to foster sustainable agriculture. The study is of prime importance because 40% of global P-deficient soil direly needs an eco-efficient solution for better cropping on a larger scale.

## Conclusion

This study provides new evidence that P-solubilizing bacteria employed beneficial impact on morpho-physiological attributes of inoculated plants leading to alleviation of P stress through induced sequential production of root exudates, modification of root architecture, and mitigation of oxidative damage by induced activities of antioxidant enzymes. These PSB-induced structural and physiological alterations in the plant ultimately led to improved wheat growth and P acquisition.

## Author Contributions

MY analyzed the data and wrote the manuscript. EI designed the setup for hydroponics. MR and NM performed data analysis and reviewed the manuscript. LR provided soils from Sindh and reviewed the manuscript. IA helped in sequencing of both 16S rRNA and gene specific amplifications. MY, MR, LR, and IF executed statistical analysis. AT analyzed HPLC results of root exudation. MI helped in the analysis of physiochemical soil characteristics. SY conceived and supervised the whole study and edited the manuscript.

## Conflict of Interest

The authors declare that the research was conducted in the absence of any commercial or financial relationships that could be construed as a potential conflict of interest.

## Publisher’s Note

All claims expressed in this article are solely those of the authors and do not necessarily represent those of their affiliated organizations, or those of the publisher, the editors and the reviewers. Any product that may be evaluated in this article, or claim that may be made by its manufacturer, is not guaranteed or endorsed by the publisher.

## Footnotes

1
https://www.ncbi.nlm.nih.gov/

2
https://blast.ncbi.nlm.nih.gov/Blast.cgi
