# Supplementary figures and images for: Differential Root Exudation and Architecture for Improved Growth of Wheat Mediated by Phosphate Solubilizing Bacteria
Source: Front Microbiol. 2021 Oct 15;12:744094. doi: 10.3389/fmicb.2021.744094 (PMC8554232; doi:10.3389/fmicb.2021.744094)

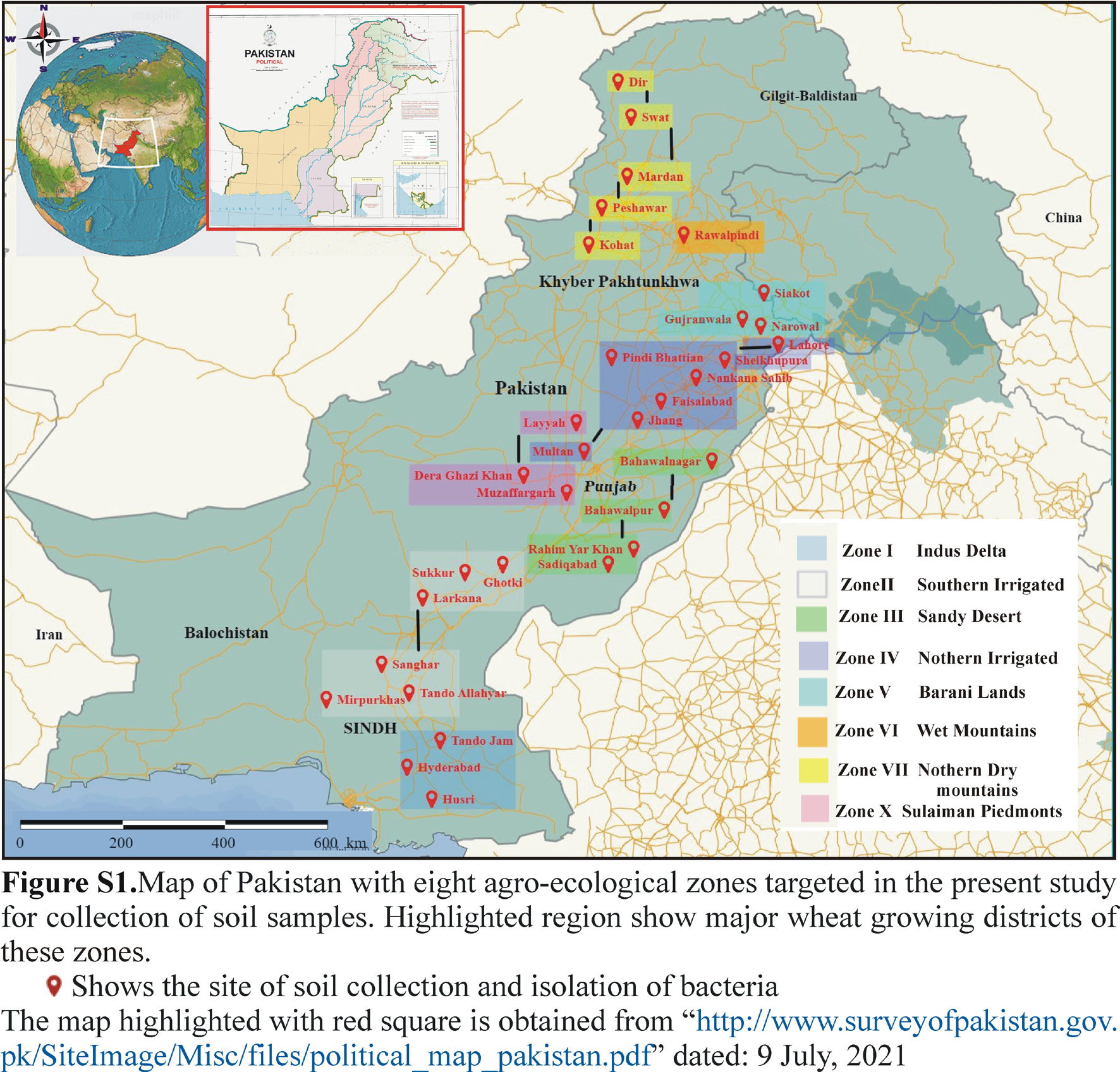

Supplement: Supplementary file 1 [file Image_1.jpg]

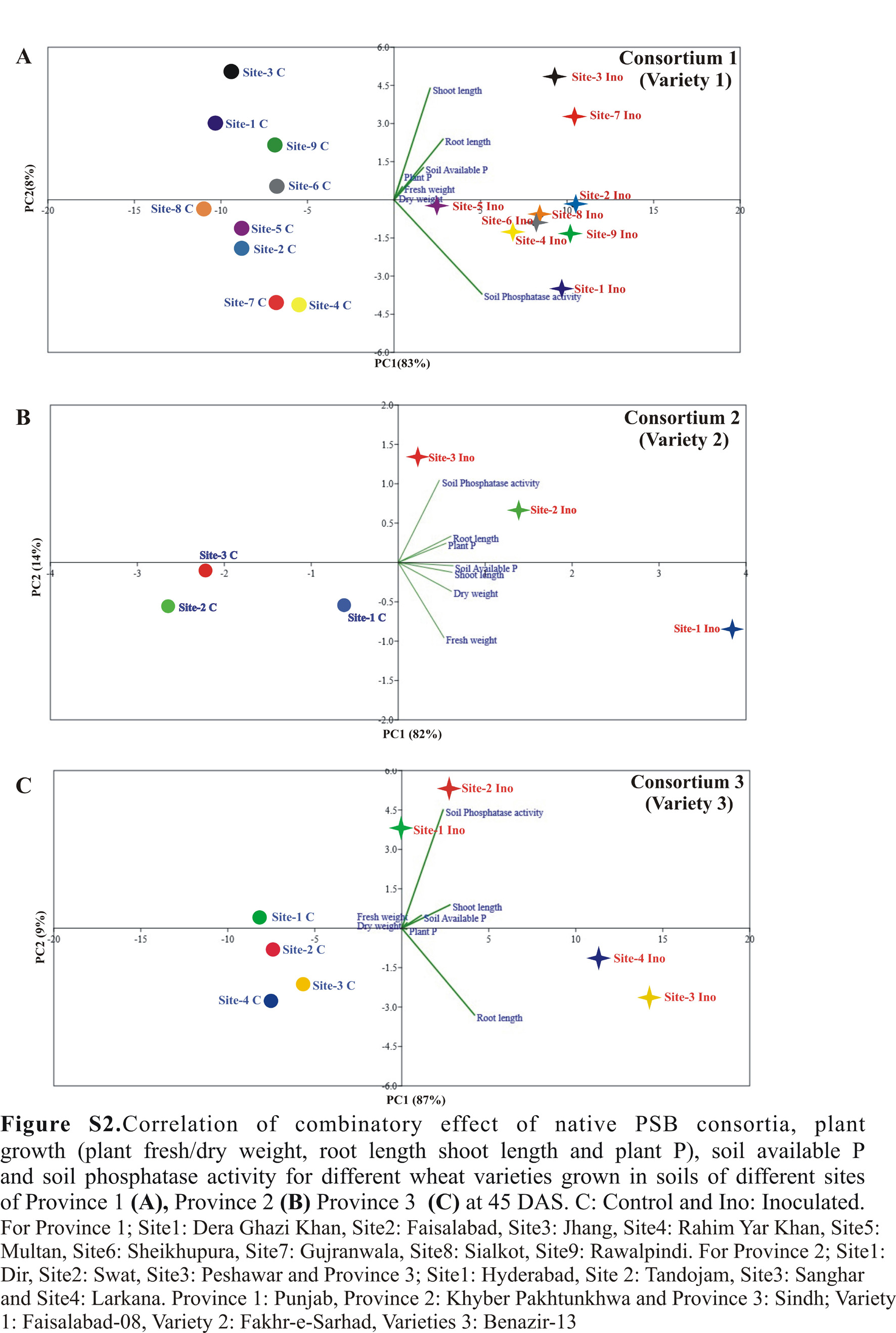

Supplement: Supplementary file 2 [file Image_2.jpg]

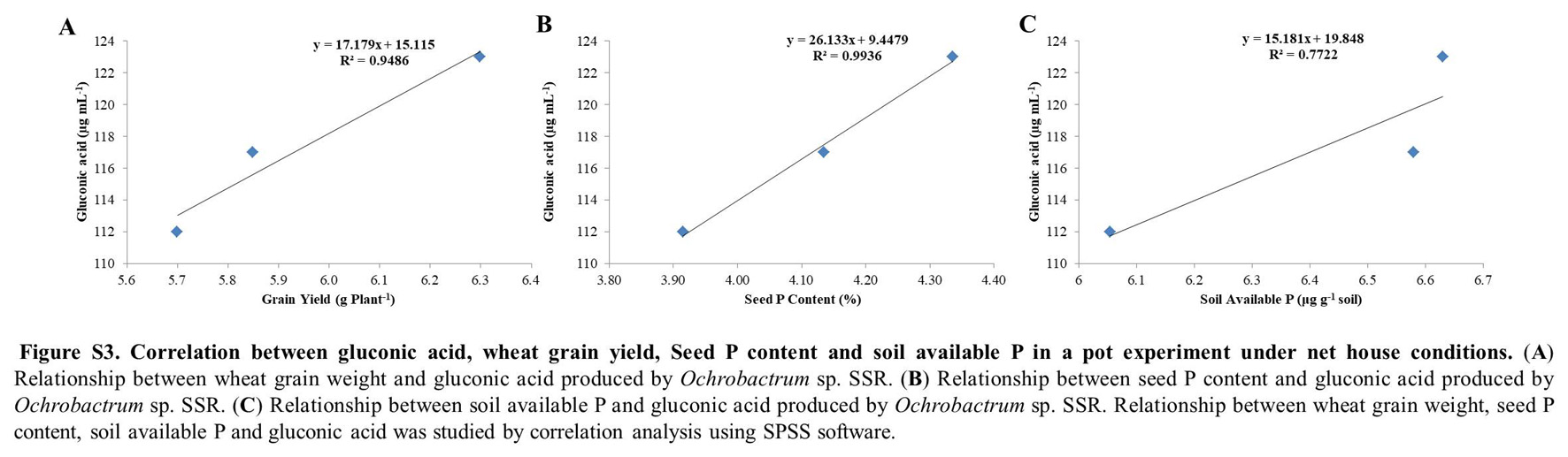

Supplement: Supplementary file 3 [file Image_3.jpg]
