# Supplementary material for: Differential Root Exudation and Architecture for Improved Growth of Wheat Mediated by Phosphate Solubilizing Bacteria
Source: Front Microbiol. 2021 Oct 15;12:744094. doi: 10.3389/fmicb.2021.744094 (PMC8554232; doi:10.3389/fmicb.2021.744094)
Supplement: Supplementary file 4 [file Data_Sheet_1.docx]

**Table S1.** Physico-chemical properties of soils collected from different field sites of wheat growing agro-ecological zones of Pakistan.

| **Province** | **Isolation Sites**  **Districts** | **Soil texture** | **pH** | **EC dSm^-1^** | **Nitrogen**  **(%)** | **Organic Matter (%)** | **Available Phosphorus (ppm)** | **Extractable Potassium (ppm)** | **Sodium**  **(ppm)** |  |  |
| --- | --- | --- | --- | --- | --- | --- | --- | --- | --- | --- | --- |
| **Province 1**  **(PUNJAB)** | Bahawalnagar | Sandy Loam | 8.34±0.42 | 1.84±0.09 | 0.02±0.002 | 0.61±0.03 | 5.89±0.29 | 157±7.85 | 449±22.45 |  |  |
|  | Bahawalpur | Sandy Loam | 8.30±0.42 | 1.74±0.09 | 0.026±0.001 | 0.50±0.03 | 5.24±0.26 | 159±7.95 | 513±25.65 |  |  |
|  | D. G. Khan | Silty Clay | 8.31±0.42 | 1.70±0.09 | 0.026±0.001 | 0.54±0.03 | 5.20±0.26 | 174±8.70 | 696±34.80 |  |  |
|  | Faisalabad | Sandy Loam | 8.26±0.41 | 1.25±0.06 | 0.03±0.002 | 0.57±0.03 | 5.82±0.29 | 153±7.65 | 512±25.60 |  |  |
|  | Hafizabad | Sandy Loam | 8.32±0.42 | 1.43±0.07 | 0.031±0.002 | 0.58±0.03 | 6.07±0.30 | 148±7.40 | 540±27.00 |  |  |
|  | Jhang | Sandy Loam | 8.35±0.42 | 1.63±0.08 | 0.028±0.001 | 0.62±0.03 | 5.85±0.29 | 138±6.90 | 641±32.05 |  |  |
|  | Gujranwala | Clay Loam | 8.38±0.41 | 1.14±0.06 | 0.029±0.001 | 0.85±0.03 | 5.85±0.29 | 156±7.80 | 458±22.90 |  |  |
|  | Lahore | Clay Loam | 8.33±0.42 | 1.69±0.08 | 0.024±0.001 | 0.79±0.04 | 6.66±0.33 | 152±7.60 | 586±29.30 |  |  |
|  | Layyah | Clay Loam | 8.23±0.41 | 1.43±0.07 | 0.031±0.001 | 0.48±0.02 | 4.44±0.22 | 110±5.50 | 293±14.65 |  |  |
|  | Nankana Sahib | Clay Loam | 8.31±0.42 | 1.51±0.08 | 0.027±0.002 | 0.58±0.03 | 7.23±0.36 | 149±7.45 | 604±30.20 |  |  |
|  | Multan | Clay Loam | 7.91±0.40 | 1.81±0.06 | 0.032±0.001 | 0.76±0.04 | 4.89±0.24 | 148±7.40 | 476±23.80 |  |  |
|  | Muzaffargarh | Clay Loam | 7.91±0.40 | 1.37±0.07 | 0.031±0.002 | 0.76±0.04 | 4.89±0.24 | 148±7.40 | 612±30.60 |  |  |
|  | Narowal | Clay Loam | 7.58±0.38 | 1.40±0.07 | 0.022±0.002 | 0.63±0.03 | 6.57±0.33 | 117±5.85 | 514±25.70 |  |  |
|  | Pindi Bhattian | Sandy Loam | 7.62±0.38 | 1.45±0.07 | 0.031±0.001 | 0.53±0.03 | 6.23±0.31 | 129±6.45 | 567±28.35 |  |  |
|  | Rahim Yar Khan | Clay Loam | 8.21±0.41 | 3.10±0.16 | 0.021±0.002 | 0.36±0.02 | 5.23±0.26 | 161±8.05 | 1153±57.65 |  |  |
|  | Sialkot | Clay Loam | 7.70±0.39 | 1.02±0.05 | 0.029±0.001 | 0.69±0.03 | 7.84±0.39 | 130±6.50 | 421±21.05 |  |  |
|  | Sheikhupura | Clay Loam | 8.28±0.41 | 3.78±0.19 | 0.023±0.001 | 0.72±0.04 | 6.34±0.32 | 163±8.15 | 1190±59.50 |  |  |
|  | Rawalpindi | Sandy Loam | 7.81±0.39 | 1.36±0.07 | 0.033±0.001 | 0.55±0.03 | 5.04±0.25 | 118±5.90 | 586±29.30 |  |  |
| **Province 2 (KPK)** | Dir | Loamy Sand | 7.3±0.37 | 1.50±0.08 | 0.026±0.001 | 0.65±0.03 | 2.5±0.13 | 152±7.60 | 595±29.75 |  |  |
|  | Kohat | Clay Loam | 7.1±0.36 | 2.75±0.14 | 0.031±0.002 | 0.45±0.02 | 5.2±0.26 | 132±9.60 | 461±23.05 |  |  |
|  | Mardan | Clay Loam | 7.3±0.37 | 1.08±0.05 | 0.028±0.001 | 0.67±0.03 | 5.4±0.27 | 181±9.05 | 403±20.15 |  |  |
|  | Peshawar | Clay Loam | 7.5±0.38 | 1.30±0.07 | 0.024±0.001 | 1.85±0.09 | 1.9±0.10 | 114±5.70 | 549±27.45 |  |  |
|  | Swat | Sandy Clay | 7.4±0.37 | 1.56±0.08 | 0.034±0.002 | 0.67±0.03 | 2.1±0.11 | 147±7.35 | 622±31.10 |  |  |
| **Province 3 (SINDH)** | Ghotki | Silty clay | 8.0±0.40 | 1.32±0.07 | 0.027±0.002 | 0.78±0.04 | 3.2±0.26 | 186±6.55 | 540±27.00 |  |  |
|  | Husri | Clay Loam | 8.2±0.41 | 2.16±0.41 | 0.017±0.001 | 0.32±0.04 | 4.3±0.22 | 181±9.05 | 1812±90.60 |  |  |
|  | Hyderabad | Clay Loam | 8.1±0.41 | 3.63±0.18 | 0.023±0.001 | 0.45±0.04 | 4.8±0.24 | 179±8.95 | 1171±58.55 |  |  |
|  | Larkana | Silty Loam | 8.0±0.40 | 3.40±0.17 | 0.029±0.001 | 0.38±0.04 | 4.7±0.24 | 156±7.80 | 668±33.40 |  |  |
|  | Mirpur Khas | Silty Loam | 8.2±0.41 | 3.09±0.15 | 0.021±0.001 | 0.26±0.04 | 3.9±0.20 | 164±8.20 | 1199±59.95 |  |  |
|  | Sanghar | Silty Loam | 8.2±0.41 | 2.90±0.15 | 0.026±0.001 | 0.19±0.04 | 3.8±0.19 | 190±9.50 | 1769±38.45 |  |  |
|  | TandoAllahyar | Clay Loam | 8.2±0.41 | 1.73±0.09 | 0.029±0.001 | 0.52±0.04 | 3.3±0.27 | 200±10.0 | 1714±35.70 |  |  |
|  | Tandojam | Clay Loam | 8.5±0.43 | 3.42±0.17 | 0.025±0.001 | 0.56±0.05 | 4.5±0.28 | 145±7.25 | 1208±60.40 |  |  |

Physico-chemical properties of soils collected from different sites of wheat growing agro-ecological zones, Pakistan. All values are an average of six biological replicates.

**Table S2:** Morpho-physiological characterization and 16S rRNA based identification of P solubilizing bacteria from different agro-ecological zones of Pakistan

| **Province** | **Phosphate Solubilizing Bacteria** | **Site of Isolation** | **GPS Coordinates^1^** | **Accession numbers** | **Colony Morphology^2^** | **Colony Picture** | **Motility** | **Gram Staining** | **Cells Morphology^3^** |
| --- | --- | --- | --- | --- | --- | --- | --- | --- | --- |
| **Province 1 (PUNJAB)** | *Enterobacter* sp. PB1 | Faisalabad | 31 ^º^ 41'8"N 73 ^º^ 07'7"E | MK422613 | Medium, smooth, shiny , circular, convex, white with entire margins | 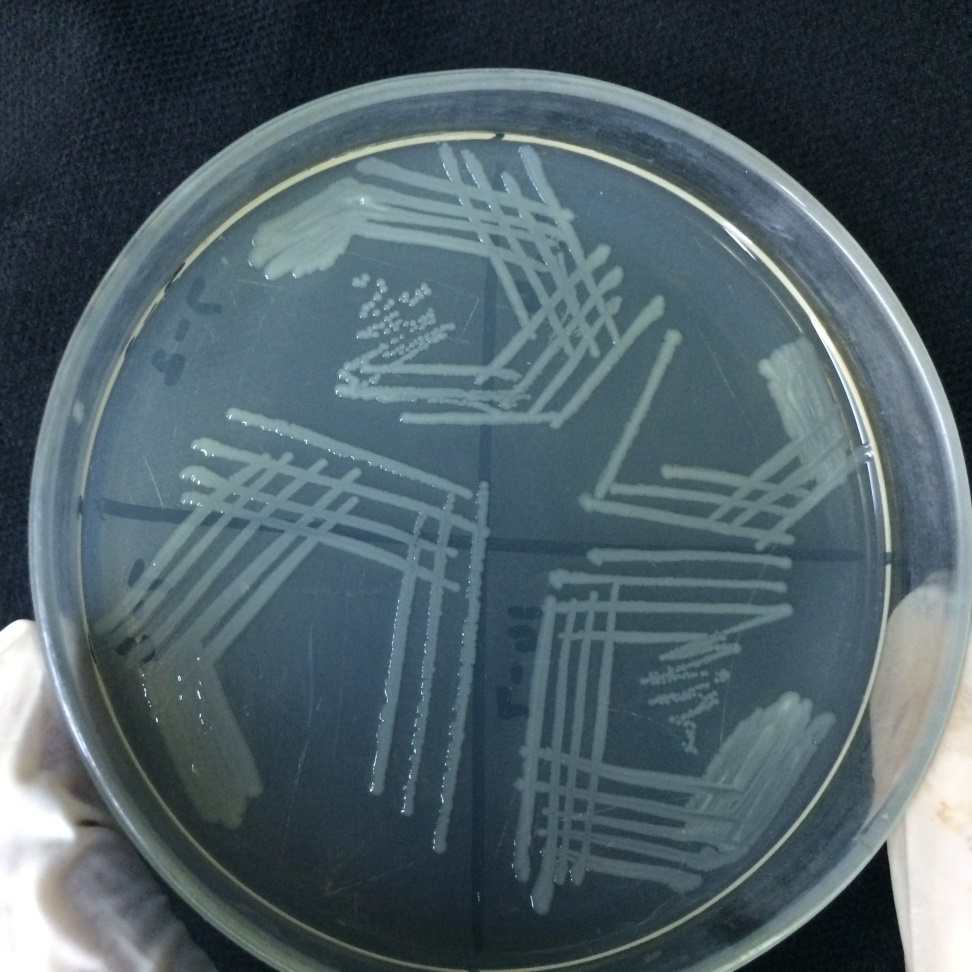 | + | - | 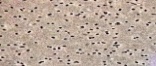 |
|  | *Enterobacter ludwigii* PB2 | Multan | 30 ^º^ 26'9"N 71 ^º^ 5'03"E | MK422614 | Small, smooth, shiny, circular, convex, off- white with entire margins | 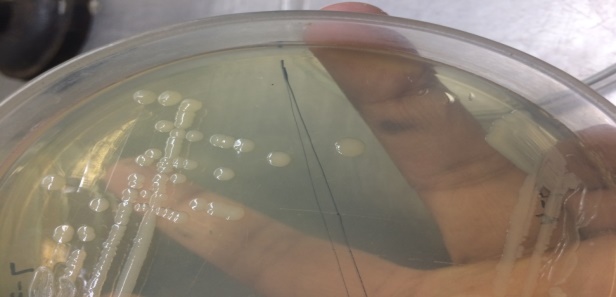 | + | - | 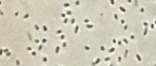 |
|  | *Enterobacter* sp. PB3 | Sheikhupura | 31^º^5'87"N 73^º^68'97"E | MK422615 | Medium, smooth, shiny , circular, raised, white with entire margins | 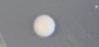 | + | - | 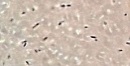 |
|  | *Stenotrophomonas* sp. PB4 | Lahore | 31^º^62'38"N 74^º^23'70"E | MK422616 | Small, smooth, shiny , circular, convex white-transparent with entire margins | 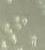 | + | - | 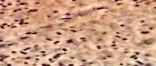 |
|  | *Enterobacter kobei* PB6 | Pindi Bhattian | 31^º^6'954"N 73^º^18'66"E | MN860098 | Medium ,smooth, shiny, circular, raised, colorless with irregular margins | 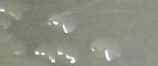 | + | - | 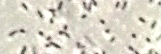 |
|  | *Stenotrophomonas maltophilia* PB147 | Rawalpindi | 33^º^6'N 73^º^0'33"E | MN860099 | Medium, smooth, shiny, circular, raised, colorless with entire margins | 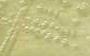 | + | **-** | 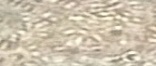 |
|  | *Ochrobactrum haematophilum* SSR * | Pindi Bhattian | 31^º^8'95"N 73^º^27'60"E | MK422612 | Medium , smooth, shiny , circular, convex, erose, white with entire margins | 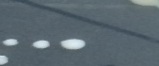 | + | - | 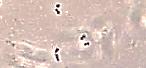 |
|  | *Enterobacter cloacae* ZW9 * | Pindi Bhattian | 31^º^8'957"N 73^º^27'60"E | MK422617 | Medium. smooth, shiny , circular, convex, erose, creamish orange with entire margins | 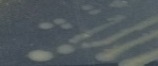 | + | - | 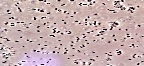 |
|  | *Enterobacter* sp. ZW32 * | Jhang | 31^º^26'94"N 72^º^3'16"E | MK817561 | Moderate, smooth, shiny , circular, convex, undulate, spread, umbonate, colorless, irregular margins | 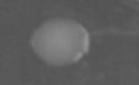 | + | - | 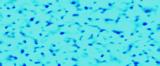 |
| **Province 2 (KPK)** | *Enterobacter cloacae* D1 * | Dir | 35^º^1'66"N 72^º^E | MK422618 | Circular, off-white, slightly raised with entire margins | 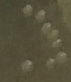 | + | - | 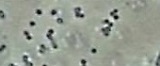 |
|  | *Enterobacter kobei* KOH | Kohat | 33^º^58'33"N 71^º^4'33"E | MN860100 | Small, smooth, shiny , lobate, raised, white | 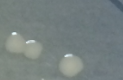 | + | - | 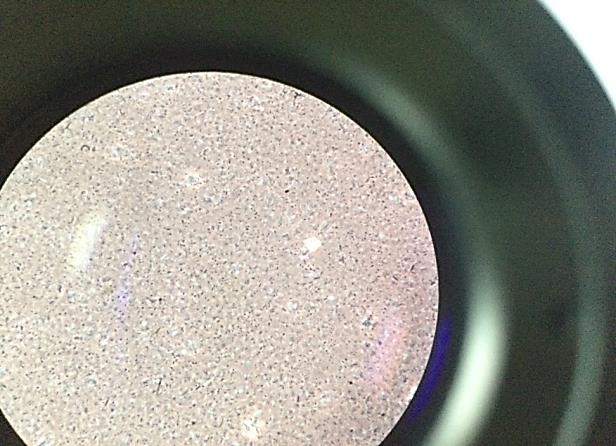 |
|  | *Pseudomonas lini* M1 | Mardan | 34^º^2'0"N 72^º^02'5"E | MN754080 | Dry, smooth, white, flat with entire margins | 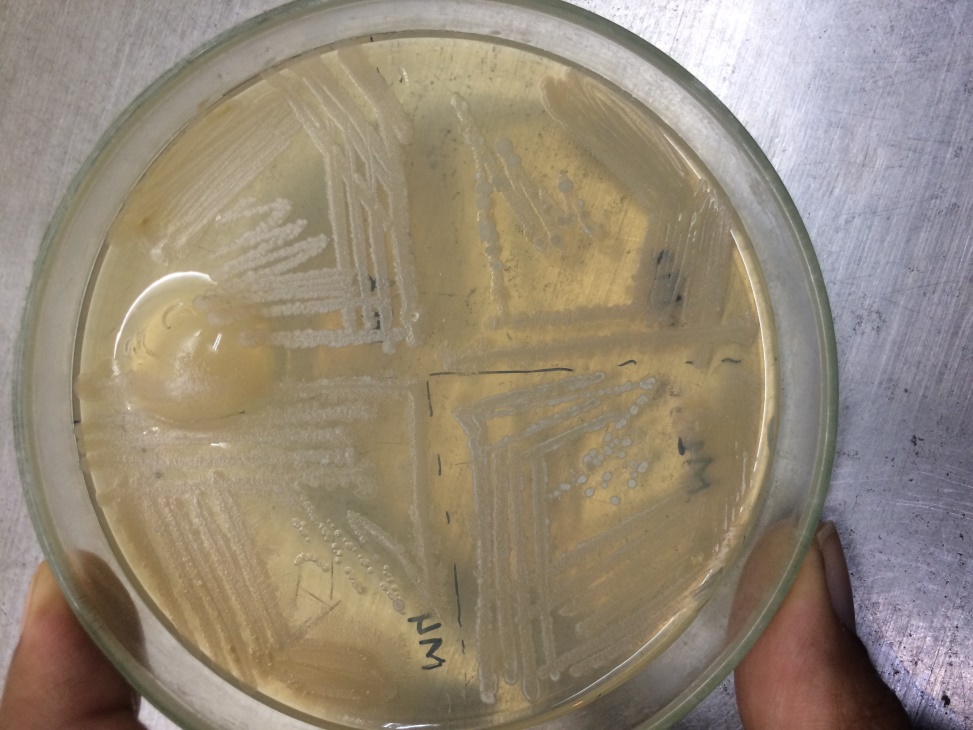 | + | **-** | 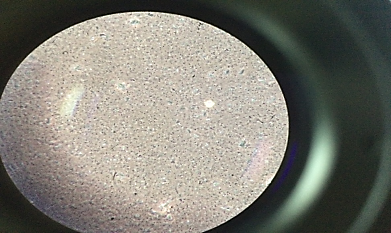 |
|  | *Acinetobacter* sp.M2 | Mardan | 34^º^20'14"N 72^º^0'2"E | MN860090 | Large, irregular, white, spread, umbonate, shiny with entire margins | 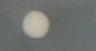 | + | **-** | 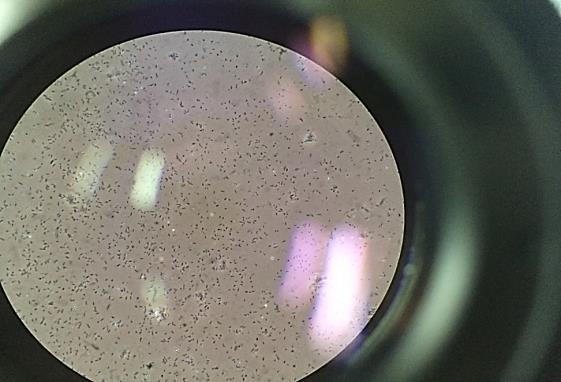 |
|  | *Pseudomonas fluorescens* M3 | Mardan | 34^º^20'22"N 72^º^0'25"E | MN860091 | Large circular, white, entire, spread, convex, shiny with entire margins | 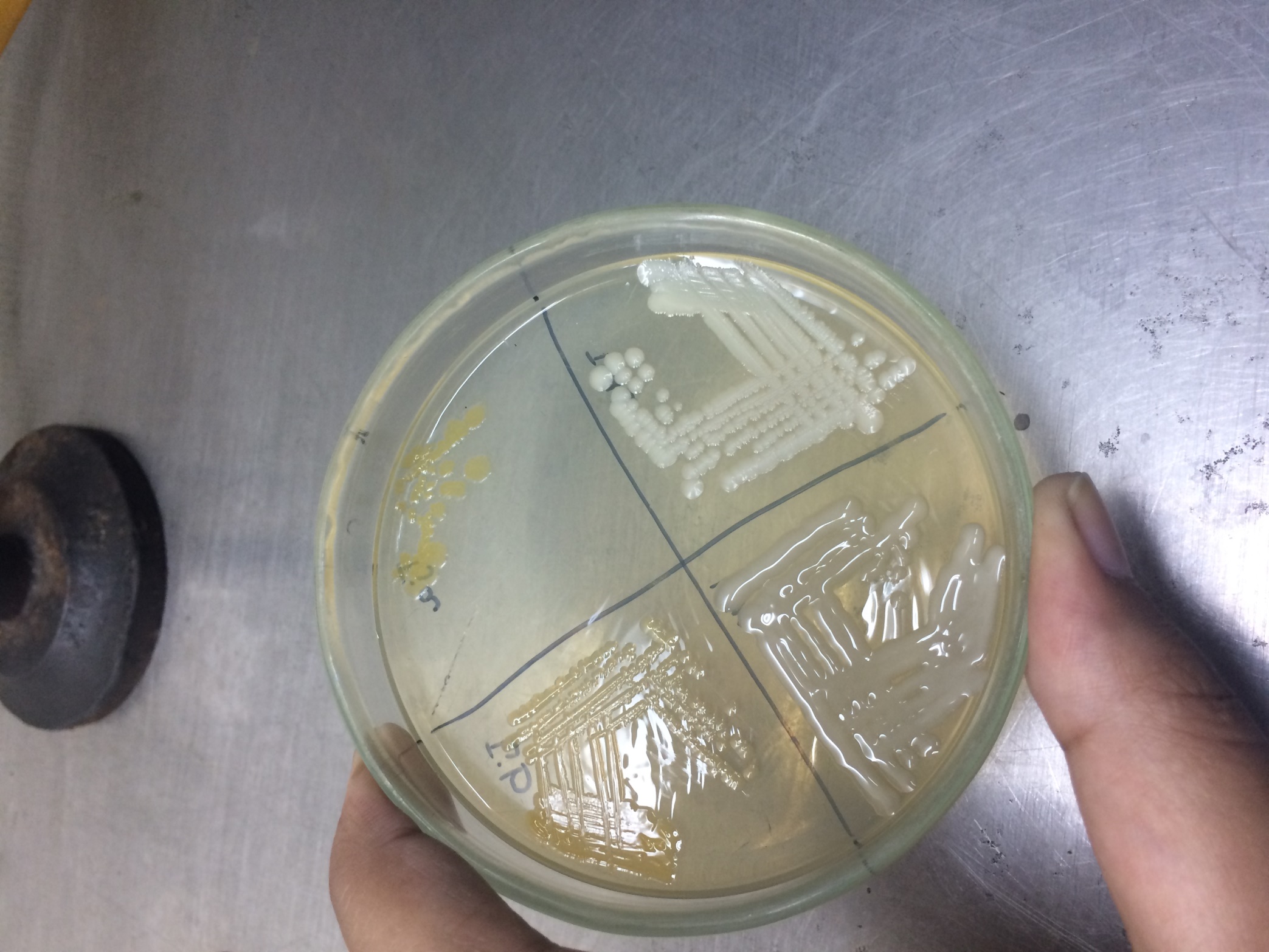 | + | **-** | 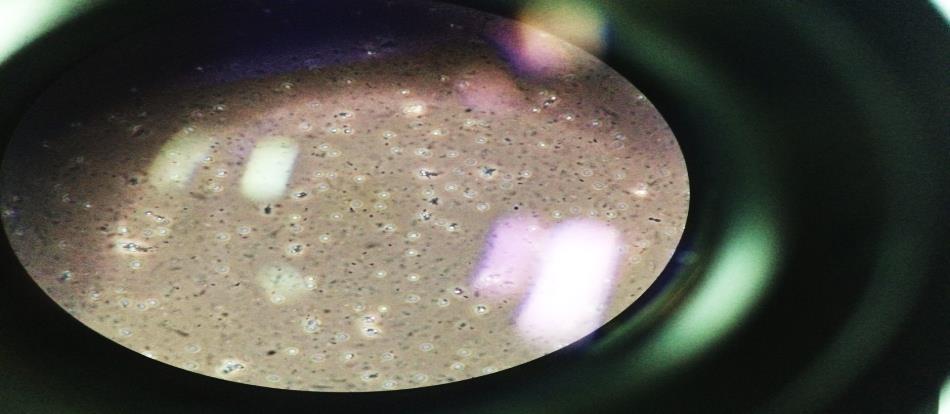 |
|  | *Pseudomonas plecoglossicida* M4 | Mardan | 34^º^40'32"N 72^º^32'44"E | MN860092 | Small, undulate, raised, shiny with irregular margins | 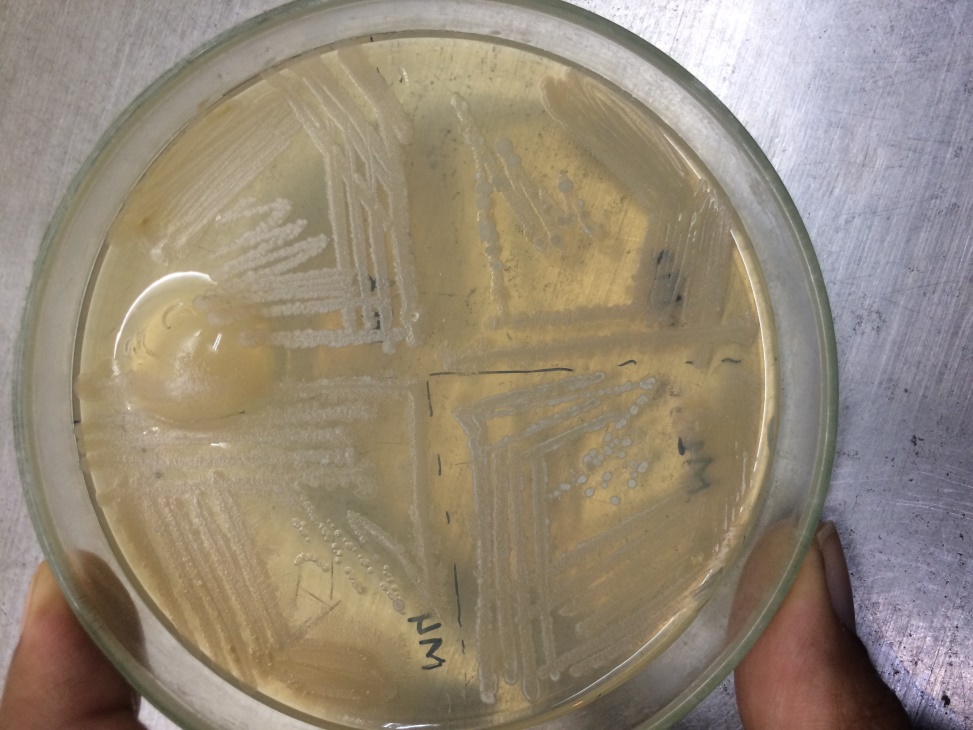 | + | **-** | 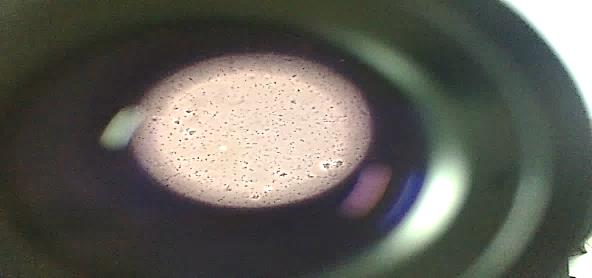 |
|  | *Pantoea agglomerans* S1 * | Swat | 35^º^38"N 72^º^1833E | MK422619 | Medium circular, raised, white-transparent, shiny with entire margins | 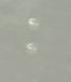 | + | **-** | 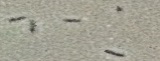 |
|  | *Enterobacter tabaci* S2 | Swat | 35^º^3'8"N 72^º^1'8"E | MN860094 | Large irregular, white, undulate, spread, slightly convex, sticky with irregular margins | 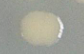 | + | **-** | 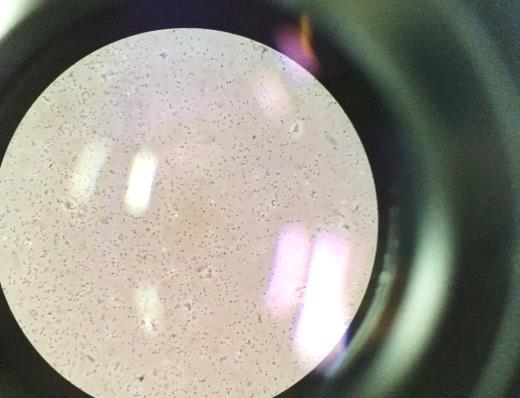 |
|  | *Pseudomonas monteilii* S5 | Swat | 35^º^0'3"N 72^º^2'5"E | MN860095 | Dull, off-white, undulate, raised, smooth, dull with irregular margins | 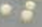 | + | **-** | 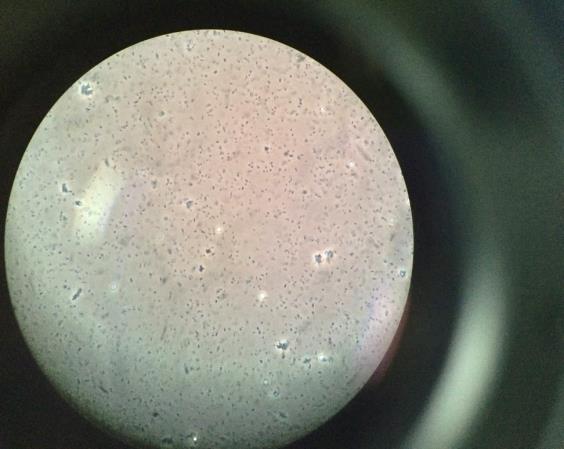 |
|  | *Bacillus* sp. S6 | Swat | 34^º^8'2" 72^º^4'8"E | MN754082 | Large, white, undulate, slightly convex, sticky with irregular margins | 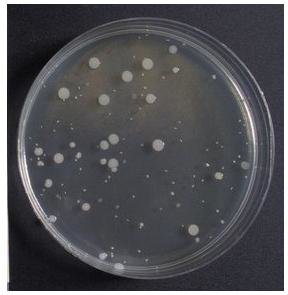 | + | + | 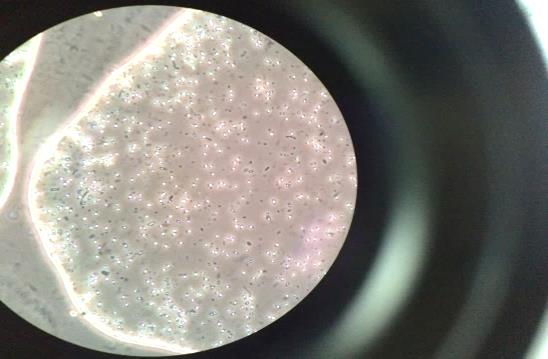 |
|  | *Enterobacter ludwigii* S7 | Swat | 34^º^6'5"N 72^º^0'3"E | MN860096 | Medium circular, white, entire, convex, smooth with entire margins | 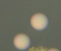 | + | **-** | 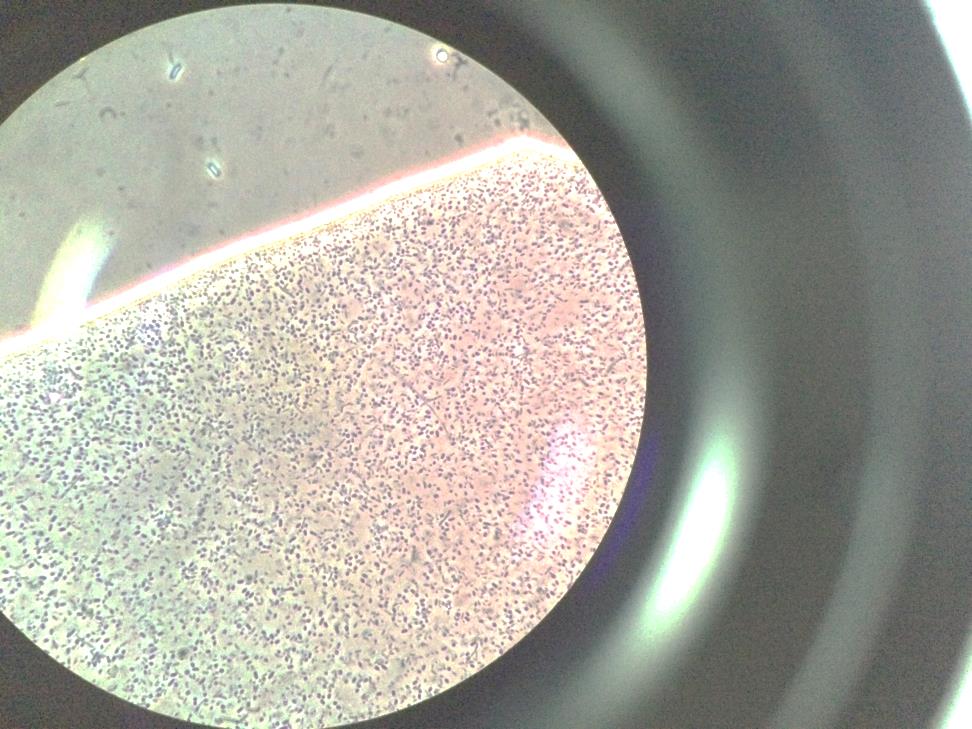 |
|  | *Enterobacter kobei* S8 | Swat | 34^º^7'3"N,72^º^0'16"E | MN860097 | Small creamish-white, shiny with entire margins | 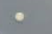 | + | **-** | 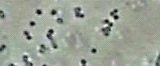 |
|  | *Pseudomonas putida* S9 | Swat | 34^º^52'19"N, 71^º^4'8"E | MK422619 | Small colorless, raised, convex, with entire margins | 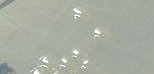 | + | - | 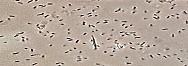 |
| **Province 3**  **(SINDH)** | *Bacillus licheniformis* TAYB * | Tando Allahyar | 25^º^4'5"N, 68^º^7'E | MN754081 | Medium, off-white, undulate, slightly raised, smooth with irregular margins | 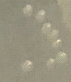 | + | + | 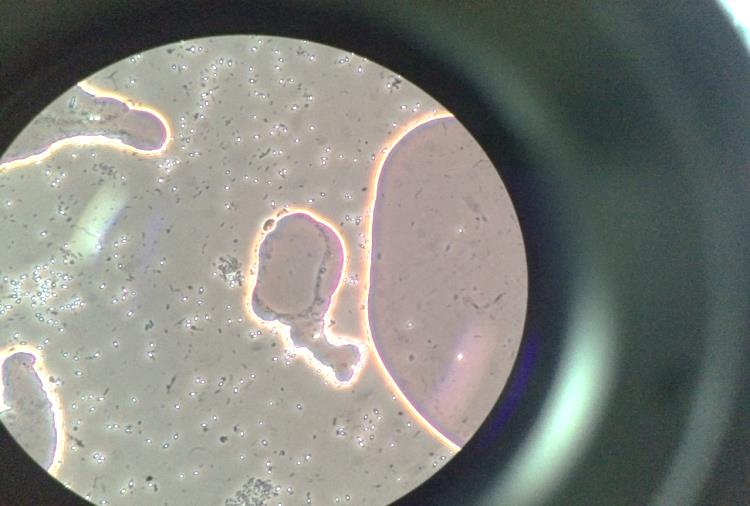 |
|  | *Pseudomonas marincola* TJA * | Tando Jam | 25^º^4'27"8N, 68^º^5'27"E | MK422620 | Medium orange, undulate, raised, rough, shiny with irregular margins | 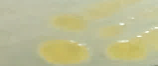 | + | - | 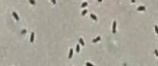 |
|  | *Enterobacter kobei* LYH1 | Larkana | 27^º^5'58"N 68^º^2'1"E | MN860102 | Medium creamish-white, lobate, raised, shiny, with irregular margins | **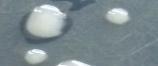** | + | - | 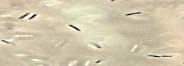 |

^1^Global Positioning System (GPS) coordinates of isolation sites were obtained by using Google Maps App.

^2, 3^ Colony and cell morphology of pure bacterial cultures were studied using light microscopy at 100X.

*Efficient PSB selected for further experiments in the present study

**Table S3:** Primers for Amplification of *Gcd, pqqE* and Phytase genes of PSB, Encoding for Proteins Involved in Organic and Inorganic Phosphate Solubilization

| **Strain** | **Gene** | **Sequence**  **5´-3´** | **Product size (bp)** | **Accession numbers** | **Homology with NCBI database at DNA level** |
| --- | --- | --- | --- | --- | --- |
| ***Enterobacter* sp. ZW32** | ***pqqE*** | F:TTYTAYACCAACCTGATCACSTC  R:TBAGCATRAASGCCTGRCG | 730 | MT897167 | 95% *Enterobacter cloaceae* [(MWMD01000001](https://www.ncbi.nlm.nih.gov/nuccore/1791116114)) |
|  | ***Phytase*** | F: ACAGACACGAAGTGACCTACC  R:CCAAGCAGACGAGAATCC | 664 | MT897166 | 94% Enterobacter ludwigii ([CP017279](https://www.ncbi.nlm.nih.gov/nuccore/1072795926)) |
| ***Ochrobactrum* sp. SSR** | ***pqqE*** | F:TTYTAYACCAACCTGATCACSTC  R:TBAGCATRAASGCCTGRCG | 725 | MT897168 | 92% [*Ochrobactrum* *pseudogrignonense* strain](https://blast.ncbi.nlm.nih.gov/Blast.cgi#alnHdr_1158824260)  (CP015776) |
| ***Pantoea* sp. S1** | ***Gcd*** | F:GACCTGTGGGACATGGACGT  R:GTCCTTGCCGGTGTAGSTCATC | 372 | MT897169 | 92% with *Pantoea* *brenneri* (CP034148) |
|  | ***Phytase*** | F:CTCGAGATGAAAGCGATCTTAATCCCAT  R:GGGAATTCATTACAAACTGCACGCCG | 962 | MT897165 | 96% with *Pantoea* *agglomerans* strain C410P1 (CP034470) |

**Table S4:**Effect of phosphate solubilizing bacteria on growth parameters of hydroponically grown wheat varieties

| **Wheat Varieties** | **Treatments** | **Fresh Weight**  **(g plant^-1^)** | **Dry Weight**  **(g plant^-1^)** | **Shoot Length (cm)** | **Plant P content (%)** |
| --- | --- | --- | --- | --- | --- |
|  |  |  |  |  |  |
| **Variety 1** |  |  |  |  |  |
|  | **Negative Control** | 0.67±0.03 D | 0.37±0.02 F | 27.56±1.25 C | 0.14±0.01 C |
|  | **Consortium-1** | 2.56±0.10 A | 0.87±0.04 A | 35.78±1.85 A | 1.57±0.08 A |
|  | **Positive Control** | 2.37±0.12 AB | 0.70±0.04 C | 31.66±1.75 B | 1.30±0.07 B |
| **Variety 2** |  |  |  |  |  |
|  | **Negative Control** | 1.43±0.07 C | 0.47±0.02 E | 32.88±1.44A B | 0.12±0.01 C |
|  | **Consortium-2** | 2.60±0.13 A | 0.73±0.04 BC | 34.86±1.74 AB | 1.57±0.08 A |
|  | **Positive Control** | 2.13±0.21 B | 0.60±0.03 D | 34.63±1.82 AB | 1.34±0.07 B |
| **Variety 3** |  |  |  |  |  |
|  | **Negative Control** | 0.73±0.04 D | 0.33±0.02 F | 34.86±1.25 AB | 0.12±0.01 C |
|  | **Consortium-3** | 2.47±0.21 A | 0.76±0.04 B | 35.40±1.59 A | 1.30±0.07 B |
|  | **Positive Control** | 2.13±0.15 B | 0.57±0.33 D | 34.63±1.65 AB | 1.29±0.06 B |

Effect of bacterial inoculation on various wheat growth parameters.Values are an average of six biological replicates. ± represent the standard deviations (SD). Means with significant difference (P<0.05) among treatments is represented by differet letters. Variety 1: Faisalabad-08, Variety 2: Fakhr-e-Sarhad, Variety 3: Benazir-13

**Table S5.** Treatments for pot experiment of different soils from different wheat zones

| **Sr. #** | **Provinve** | **Site#** | **Soil collection site** | **Treatments** | **Discription** | **Treatments** | **Discription** |
| --- | --- | --- | --- | --- | --- | --- | --- |
|  | **Province 1**  **(PUNJAB)** | **1** | DG Khan | T1 | Inoculated soil | T17 | Un-inoculated soil |
|  |  | **2** | Faisalabad | T2 | Inoculated soil | T18 | Un-inoculated soil |
|  |  | **3** | Jhang | T3 | Inoculated soil | T19 | Un-inoculated soil |
|  |  | **4** | Multan | T4 | Inoculated soil | T20 | Un-inoculated soil |
|  |  | **5** | Rahim Yar Khan | T5 | Inoculated soil | T21 | Un-inoculated soil |
|  |  | **6** | Sheikhupura | T6 | Inoculated soil | T22 | Un-inoculated soil |
|  |  | **7** | Gujranwala | T7 | Inoculated soil | T23 | Un-inoculated soil |
|  |  | **8** | Sialkot | T8 | Inoculated soil | T24 | Un-inoculated soil |
|  |  | **9** | Rawalpindi | T9 | Inoculated soil | T25 | Un-inoculated soil |
|  | **Province 2 KPK** | **1** | Dir | T10 | Inoculated soil | T26 | Un-inoculated soil |
|  |  | **2** | Swat | T11 | Inoculated soil | T27 | Un-inoculated soil |
|  |  | **3** | Peshawar | T12 | Inoculated soil | T28 | Un-inoculated soil |
|  | **Province 3 SINDH** | **1** | Hyderabad | T12 | Inoculated soil | T29 | Un-inoculated soil |
|  |  | **2** | TandoJam | T14 | Inoculated soil | T30 | Un-inoculated soil |
|  |  | **3** | Sanghar | T15 | Inoculated soil | T31 | Un-inoculated soil |
|  |  | **4** | Larkana | T16 | Inoculated soil | T32 | Un-inoculated soil |

**Table S6:** Soil analysis to study the effects of PSB inoculation on different wheat varieties grown in soils of different sites

|  |  | | **Province 1** | | |  | | | **Province 2** | | **Province 3** | | |  |
| --- | --- | --- | --- | --- | --- | --- | --- | --- | --- | --- | --- | --- | --- | --- |
|  | **Soil Sites** | **Soil Available P (μg g^-1^ soil)** | | **Soil Phosphatase**  **Activity (μmoles g^-1^ soil hr^-1^)** | **Viable**  **(CFU g-^1^soil)** | **Soil Sites** | **Soil Available P (μg g^-1^ soil)** | **Soil Phosphatase**  **Activity (μmoles g^-1^ soil hr^-1^)** | | **Viable**  **(CFU g-^1^soil)** | **Soil Sites** | **Soil Available P (μg g^-1^ soil)** | **Soil Phosphatase**  **Activity (μmoles g^-1^ soil hr^-1^)** | **Viable**  **(CFU g-^1^soil)** |
| **INOCULATED PLANTS** | **Site 1** | 5.24±0.24 D | | 29.62±1.48 A | 3.2 x 10^7^ | **Site 1** | 9.49±0.38 A | 26.79±1.75 B | | 3.9 x 10^7^ | **Site 1** | 4.21±0.18 C | 17.00±0.62 C | 4.2 x 10^7^ |
|  | **Site 2** | 5.44±0.24 CD | | 28.43±1.41 AB | 8.8 x 10^7^ | **Site 2** | 5.94±0.45 B | 25.86±1.63 B | | 4.8 x 10^7^ | **Site 2** | 4.00±0.20 C | 20.76±1.07 A | 4.3 x 10^7^ |
|  | **Site 3** | 7.20±0.35 B | | 24.33±1.04 D | 7.6 x10^7^ | **Site 3** | 5.09±0.20 C | 30.03±0.97 A | | 6.66 x10^7^ | **Site 3** | 6.46±0.33 A | 19.20±0.92 B | 7.6 x10^7^ |
|  | **Site 4** | 4.38±0.20 E | | 26.33±-1.36 C | 5.9 x 10^7^ |  |  |  | |  | **Site 4** | 4.61±0.39 B | 18.00±0.90 C | 4.6 x 10^7^ |
|  | **Site 5** | 5.31±0.27 CD | | 22.50±1.51 E | 4.9 x 10^7^ |  |  |  | |  |  |  |  |  |
|  | **Site 6** | 5.49±0.32 CD | | 27.00±1.32 BC | 3.3 x 10^7^ |  |  |  | |  |  |  |  |  |
|  | **Site 7** | 9.82±0.46 A | | 27.60±1.28 BC | 8.5 x 10^7^ |  |  |  | |  |  |  |  |  |
|  | **Site 8** | 5.31±0.20 CD | | 27.14±1.21 BC | 7.4 x10^7^ |  |  |  | |  |  |  |  |  |
|  | **Site 9** | 5.63±0.26 C | | 26.59±1.34 C | 4.4 x 10^7^ |  |  |  | |  |  |  |  |  |
| **UN-INOCULATED CONTROLS** | **Site 1** | 1.07±0.05 K | | 10.25±0.52 IJ | 0.7 x 10^7^ | **Site 1** | 2.79±0.27 D | 13.24±0.57 C | | 1.3 x 10^7^ | **Site 1** | 1.56±0.05 D | 12.27±0.64 D | 1.0 x 10^7^ |
|  | **Site 2** | 1.52±0.04 GHI | | 15.07±0.89 G | 1.0 x 10^7^ | **Site 2** | 1.50±0.08 F | 12.38±0.54 CD | | 0.8 x 10^7^ | **Site 2** | 1.71±0.08 D | 11.20±0.61 D | 1.2 x 10^7^ |
|  | **Site 3** | 1.42±0.04 HIJ | | 9.70±0.44 J | 0.6 x10^7^ | **Site 3** | 2.59±0.19 E | 10.85±0.75 D | | 0.6 x10^7^ | **Site 3** | 1.54±0.02 D | 9.93±0.50 E | 0.6 x10^7^ |
|  | **Site 4** | 1.17±0.06 JK | | 18.27±0.92 F | 0.4 x 10^7^ |  |  |  | |  | **Site 4** | 1.85±0.05 D | 9.67±0.47 E | 0.4 x 10^7^ |
|  | **Site 5** | 2.45±0.22 GHI | | 14.30±0.60 GH | 0.9 x 10^7^ |  |  |  | |  |  |  |  |  |
|  | **Site 6** | 1.24±0.05 IJK | | 14.38±0.68 GH | 0.3 x 10^7^ |  |  |  | |  |  |  |  |  |
|  | **Site 7** | 1.76±0.05 GH | | 16.95±0.93 F | 1.3 x 10^7^ |  |  |  | |  |  |  |  |  |
|  | **Site 8** | 1.84±0.07 G | | 11.47±0.50 I | 0.5 x10^7^ |  |  |  | |  |  |  |  |  |
|  | **Site 9** | 1.43±0.07 HJJ | | 13.35±0.65 H | 0.7 x 10^7^ |  |  |  | |  |  |  |  |  |

Effect of bacterial inoculation on soil parameters at 45 DAS: Days after sowing.Values are an average of 6 biological replicates. ± represent the standard deviations (SD). Means with significant difference (P<0.05) among treatments is represented by different letter. For **Province 1**; Site1: Dera Ghazi Khan, Site2: Faisalabad, Site3: Jhang, Site4: Rahim Yar Khan, Site5: Multan, Site6: Sheikhupura, Site7: Gujranwala, Site8: Sialkot, Site9: Rawalpindi. For **Province 2;** Site1: Dir, Site2: Swat, Site3: Peshawar and **Province 3;** Site1: Hyderabad, Site 2: Tandojam, Site3: Sanghar and Site4: Larkana. Province 1: Punjab, Province 2: Khyber Pakhtunkhwa and Province 3: Sindh.
